# The snow meteorology and phenology classification (SnowMAP): global snow cover observations enhance snow’s representation

**DOI:** 10.1038/s41598-026-44321-x

**Published:** 2026-03-18

**Authors:** Jeremy Johnston, Jennifer M. Jacobs, Megan Vardaman, Eunsang Cho

**Affiliations:** 1 Earth Systems Research Center, University of New Hampshire, Durham, NH USA; 2 Department of Civil and Environmental Engineering, University of New Hampshire, Durham, NH USA; 3 Ingram School of Engineering, Texas State University, San Marcos, TX USA

**Keywords:** Climate sciences, Environmental sciences

## Abstract

Snow is a vital water resource that regulates climate, supports ecosystems, and influences economies and transportation systems, making it essential to understand its physical properties and the seasonal timing of its presence (phenology). However, current snow classifications separate snow’s meteorological controls from its phenology, limiting its decision-relevance. This study introduces SnowMAP, a global snow classification system that combines snow meteorological and phenology classes. By integrating meteorological factors such as snowfall, temperature, and wind with seasonal dynamics like snow presence and melt timing, SnowMAP provides a more complete view of global snow conditions. The resulting 18 snow classes reflect notable variations in snow depth, geography, land cover, and infrastructure. SnowMAP provides a unified framework for describing how a snowpack forms and evolves, enabling scientists, planners, and communities to better understand snow conditions and their impact on the natural and built environments.

**Supplementary Information:**

The online version contains supplementary material available at 10.1038/s41598-026-44321-x.

## Introduction

Snow properties, such as its depth, density, or water content, are challenging to measure because they vary throughout the vertical profile of a snowpack, over short distances, and can change in a matter of hours^1–3^. Still, observing and modeling these properties remains critical, as they influence water availability^4,5^, flood hazards^6,7^, global climate and local meteorological conditions^8–11^, ecological processes like plant growth^12,13^, as well as the economy through impacts to transportation systems and recreation^14–17^. In the face of uncertainty, classification systems enable complex variables like snow to be condensed into a brief and interpretable code, allowing users to extrapolate information beyond what is directly measured^18^. For example, plant hardiness zones^19^ provide a simplified summary of extreme temperatures that gardeners widely apply to understand where certain plants are likely to thrive, while engineers rely on hazard maps of the 20, 50, and 100-year floodplains to identify areas with a high risk of flooding^20^.

To classify the properties of snow on a global scale, Sturm et al. (1995)^18^ published the *‘Seasonal Snow Cover Classification System’*, drawing on decades of field experience and observations. They used long-term air temperature and precipitation datasets^21,22^, as well as wind exposure data, to partition the globe into six snow classes: ephemeral, maritime, alpine, prairie, tundra, and taiga. In their classification, land cover type (forested vs. non-forested) provided a proxy for wind exposure (low vs. high wind). A decision tree was used to combine meteorological and proxy wind variables to best represent various snowpack properties observed in the field and to define each snow class. For example, tundra snow (low air temperature, low precipitation, high wind exposure) is described as a shallow, cold, wind-blown snowpack forming dense wind slabs and drifts, driving high spatial variability in snow density. Alternatively, ephemeral snow (very high air temperature) was described as a shallow, warm, and wet snow (near 0 °C) that melts soon after it accumulates. Extensive research since Sturm et al. (1995)^18^ published their work has confirmed the dominant drivers of snow variability to be meteorology, vegetation, terrain, and their interactions across spatial scales^23–25^. In 2021, Sturm and Liston (2021)^26^ updated their classification approach with enhanced spatial resolution data (i.e., from hundreds of kilometers to sub-kilometer scales), updated precipitation and temperature thresholds, and updated class naming conventions, all based on a recent period (1981–2019). This classification system is hereafter referred to as the *meteorological snow classes*^26^.

In recent decades, the availability of global-scale satellite observations with high spatiotemporal resolution (e.g., MODIS, launched in late 1999) has enabled reliable and consistent observations of snow cover^27^, and in turn, a record of snow accumulation and melt timing not captured by meteorological snow classes. Snow cover records have been used to link snow cover to seasonal runoff processes^28,29^ and vegetation green-up^30^ as well as to identify significant reductions in the extent and duration of snow cover^31,32^. These applications rely on snow persistence, or the duration that snow cover lasts after snowfall, to provide insights into the seasonal dynamics of snowpacks, referred to as their ‘seasonality’ or ‘phenology’. Such observations are distinct from the long-term climatological averages driving meteorological snow classes because they represent the temporal variability of snow conditions.

To classify snow using a phenological approach, global snow cover records (2000–2023) from the Moderate Resolution Imaging Spectroradiometer (MODIS) were utilized to summarize snow cover patterns over multiple decades^33^. In the Johnston et al. (2023)^33^ snow phenology classification, hereafter referred to as the *snow phenology classes*, global land areas are categorized into five classes: perennial (i.e., permanent), seasonal, transitional, ephemeral, or no snow (i.e., no observed snow cover in most years). The three intermediate phenology classes in Johnston et al., (2023)^33^ represent the annual cycling of snowpacks, where:

1. Seasonal snow has a persistent, long-lasting snowpack (more than six months of continuous snow cover) and a single distinct snow accumulation and melt period.
2. Transitional snow has an extended period of snow cover (generally two to six months of total snow cover), but with multiple melt and accumulation cycles.
3. Ephemeral snow has multiple short periods of snow cover (generally less than two months in total).

These snow phenology classes have been used to characterize regions by the sub-seasonal cycling of snow accumulation and melt^34–36^.

Since 1995, both the snow and broader scientific communities have widely adopted the meteorological snow classification system^18^. Included in hundreds of published manuscripts, the snow classification system has fostered the growth of organized, targeted snow research, allowing for studies to be linked across mountain ranges, regions, and continents into shared domains of relevance (e.g., tundra, ephemeral snow). The Sturm et al. (1995) snow classification has been predominantly used to characterize study areas or individual sampling locations^26^. Many studies report the meteorological snow classes of their domain or sampling sites to (1) allow the reader to better understand the environment (or data) that the study was performed in (or with) by associating it with expected snow conditions, such as deep snow, wet snow, ice layers^37^, and (2) to infer physical or statistical properties of snowpacks in different regions that are challenging to observe or model, like density or depth^38,39^. Snow classifications have also guided snow remote sensing methods^40,41^ and been used to understand their performance^35,42^. These applications rely on knowledge of each class’s underlying land surface and snow characteristics to describe study areas, develop research questions, plan field activities such as NASA SnowEx campaigns (https://snow.nasa.gov/campaigns/snowex), and evaluate and parameterize models and remote sensing algorithms^43,44^.

**Fig. 1:**
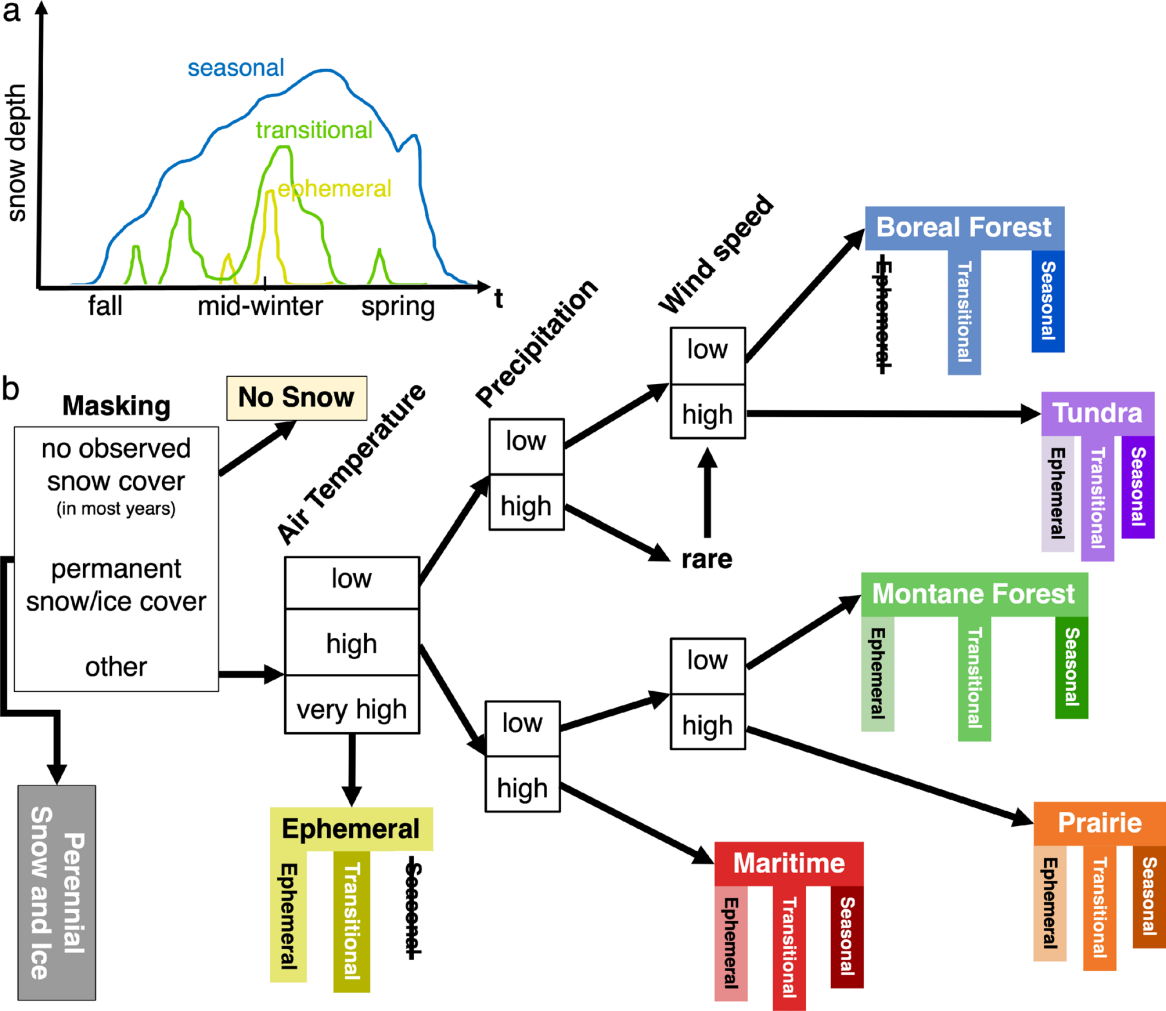
The SnowMAP (Snow Meteorology and Phenology) classification framework combines Sturm and Liston’s 2021 meteorological snow classification and Johnston et al., 2023 snow cover phenology (i.e., seasonality) classes. (**a**) Shows a simplified illustration of the snow phenology classes (modified from Fig. 1 in Johnston et al., 2023), while (**b**) shows the SnowMAP classification framework (modified from the meteorological snow classification decision trees in Sturm et al., 1995 and Sturm and Liston, 2021). A total of 18 global classes were identified by partitioning global land areas by snow cover duration, snow on/off cycles, temperature, precipitation, and wind speed (forested vs. non-forested). Classes not found in nature are struck through, indicated by crossed-out seasonality classes (e.g., ephemeral boreal forest). Very cold regions with high precipitation are rare on Earth, as noted in Sturm et al., 1995 and Sturm and Liston 2021. These classes are thus merged with the Boreal Forest and Tundra snow classes.

Despite their utility, meteorological classes alone do not capture when snow arrives, how long it persists, or how rapidly it melts. In a warming climate—marked by earlier melt onset, shorter seasons, shifts in the rain–snow transition, and more mid-winter thaws^4,5,31,33^—these phenological dimensions increasingly govern hydrological and societal impacts. A framework that integrates phenology alongside meteorological snow types is therefore necessary to evaluate change, compare regions consistently, and develop decision-relevant indicators. To address these gaps, this work demonstrates the differences between snow properties (i.e., meteorological classes) and snow persistence (i.e., phenological classes) classifications, showing that when used in combination, a more complete picture of winter snow conditions emerges. This combined global snow classification system is illustrated in Fig. 1 and referred to as the *Snow Meteorology and Phenology (SnowMAP)* classes (specific thresholds are detailed in Supplementary Table 1). In the following sections, we first summarize the SnowMAP classes and distinguish them from the widely used Sturm and Liston (2021)^26^ meteorological snow classes. We then illustrate the value of SnowMAP in characterizing snow depth variability. We next define the geographic controls, the land cover, and land use characteristics of each SnowMAP class. The results also briefly highlight two use cases for the new classification system and conclude by discussing the broader relevance and future uses for the SnowMAP snow classification dataset, available at 10.5281/zenodo.17373632.

## Results

### The global distribution of SnowMAP classes

**Fig. 2:**
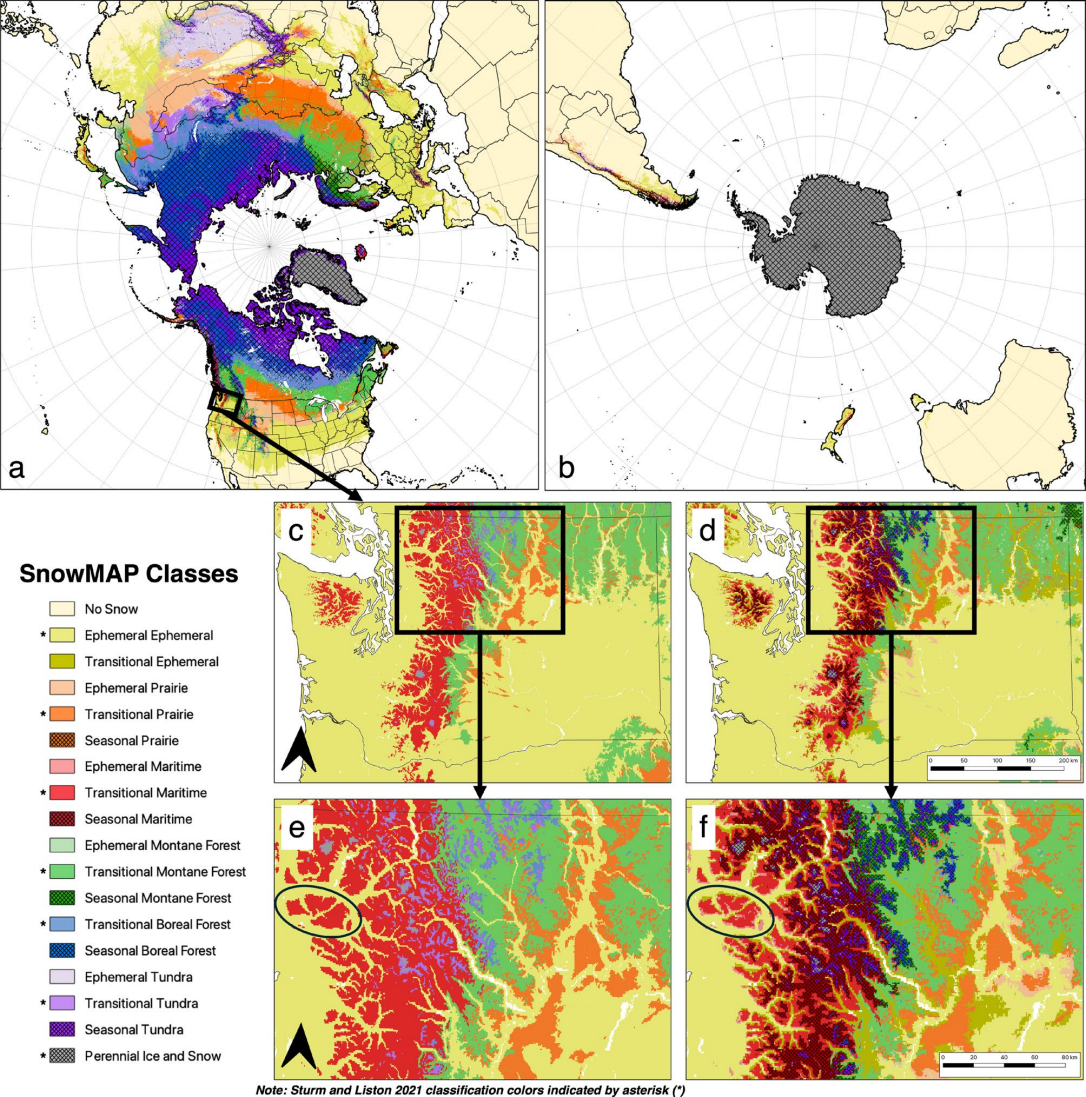
The addition of snow seasonality classes provides new insights into snowpack variability across spatial scales. (**a**) The SnowMAP (Snow Meteorology And Phenology) combined snow classification for the Northern Hemisphere (inset is the U.S. state of Washington) and (**b**) the Southern Hemisphere. Grid lines are spaced at 10° increments for latitude and longitude. Panels (**c**) and (**e**) show the meteorological snow classes (Sturm and Liston 2021) for Washington and zoomed in to the Cascade Range in north central Washington, respectively. Panels (**d**) and (**f**) show the same areas as in (c) and (e), but seasonal and perennial snow classes are indicated by black hatches/darker shades, while ephemeral regions are depicted with lighter shades. A darker shade was introduced for the ‘Transitional Ephemeral’ class; all other transitional areas remain unchanged from the coloring in panels (c) and (e). Colors used for panels (c) and (e) are indicated by an asterisk (*).

Snow phenology provides timing information that refines each meteorological snow class, yielding 18 integrated SnowMAP classes, which are summarized by their spatial extents and definitions in Table 1. Note that for SnowMAP classes, snow phenology classes are used as a modifier (or sub-class) to each established meteorological snow class (e.g., transitional prairie) and to differentiate between ‘no snow’ areas that do not receive observable snow in most winters from those that do. With this approach, the widely used snow property descriptions detailed in the meteorological snow classifications^18,26^ remain unchanged, and the temporal component of when to expect such conditions is added via the snow phenology classes. Perennial (i.e., permanent) ice and snow classes are combined. Figure 2 provides global maps of the SnowMAP classes, which combine meteorological and phenological characteristics, by hemisphere (a-b), with sub-panels (c-f) highlighting details observed with the 30 arc second (~ 1 km) dataset. No snow areas covered the largest extent of any SnowMAP class globally, over 50% of all land areas (76 million km^2^). When excluding no snow and perennial ice and snow regions (e.g., Antarctica, Greenland), areas receiving snowfall in most winters are overwhelmingly located in the Northern Hemisphere (52.7 million km^2^) relative to the Southern Hemisphere (1.5 million km^2^) (Fig. 2a,b). Asia, North America, and Europe include a diverse range of snow climates in contrast to South America, Australia/Oceania, and Africa, which are primarily classified as no snow (see Supplementary Fig. 1).

As shown in Table 1, four of the six meteorological snow classes, excluding ice, have multiple snow phenology sub-classes covering a large percentage of their area (prairie, maritime, boreal forest, and tundra). Globally, more than 98% of prairie snow is classified as either ephemeral (42%) or transitional (56%), indicating that these high-wind areas, which are both moisture- and cold-limited, rarely develop persistent seasonal snowpacks. Within maritime snow classes, characterized by high precipitation and milder temperatures, steep elevation gradients drive temperature variability and the development of three snow phenology classes, which cover a significant portion of these regions (exceeding 24% for each sub-class). In contrast, the montane forest class is predominantly classified as transitional (78%) with approximately 10% coverage of both ephemeral and seasonal snow sub-classes. In the boreal forest, only transitional and seasonal sub-classes occur. While seasonal snow lasting more than 6 months is most common in boreal forest classes (69%), a shift towards less persistent transitional snowpacks occurs at lower latitudes (Fig. 2a). Tundra regions include all three phenology classes but are predominantly seasonal (70%). A unique class, the ephemeral tundra, covering 16% of the tundra snow class, is prevalent in cold, dry, and windy regions, such as the Tibetan Plateau of High Mountain Asia (Fig. 2a). The only case with high overlap between the snow classifications is for ephemeral regions, as 98% of those classified as ephemeral in the meteorological classification are also classified as ephemeral in the snow phenology classification (‘ephemeral ephemeral’).

**Table 1:** Descriptions of each SnowMAP (Snow Meteorology and Phenology) class resulting from combining the meteorological and snow phenology classifications, including the land area covered by each class globally, and by hemisphere. Notes on the snow properties from each meteorological snow class (via Sturm et al. 1995; Sturm and Liston 2021) and seasonality characteristics (via Johnston et al., 2023) are included in the description. No snow and perennial snow areas have been excluded from the column totals to highlight the spatial coverage of the snow phenology classes (ephemeral, transitional, and seasonal). Together, the no snow and perennial classes account for 63% of global land areas (53% no snow, 10% perennial snow).

Meteorological snow class (Sturm and Liston 2021)	Snow phenology class (Johnston et al., 2023)	Land area in millions (M) of km^2^ (% of column total)			Description
		Global	Northern Hemisphere	Southern Hemisphere	
Ephemeral	No snow	76.00 M·km^2^	43.20 M·km^2^	32.80 M·km^2^	No observed snow cover in most winters
Ephemeral	Ephemeral	12.20 M·km^2^ (22%)	11.43 M·km^2^ (22%)	0.77 M·km^2^ (53%)	Generally shallow (< 0.5 m), seasonally discontinuous snow cover with dense, wet snow from melting soon after snowfall. Lasting for < 2 months total in most winters. Transitional areas have individual winters with snow cover lasting continuously for 2—4 months
	Transitional	0.31 M·km^2^ (< 1%)	0.27 M·km^2^ (< 1%)	0.04 M·km^2^ (3%)	
Prairie	Ephemeral	4.17 M·km^2^ (8%)	3.93 M·km^2^ (7%)	0.25 M·km^2^ (17%)	High variability in snow depth and density due to wind drifting. Little to no forest cover. Milder temperatures as compared to tundra. Most commonly classified as ephemeral areas with discontinuous snow cover lasting < 2 months or transitional areas with sustained snow cover for 2—4 months in most winters. Seasonal prairie is rare
	Transitional	5.64 M·km^2^ (10%)	5.61 M·km^2^ (11%)	0.03 M·km^2^ (2%)	
	Seasonal	0.19 M·km^2^ (< 1%)	0.19 M·km^2^ (< 1%)	< 0.01 M·km^2^ (< 1%)	
Maritime	Ephemeral	0.18 M·km^2^ (< 1%)	0.05 M·km^2^ (< 1%)	0.13 M·km^2^ (9%)	Generally deep and dense snow which may exceed 5 m in wet years. Characterized by high precipitation and mild temperatures, which results in a wide range of precipitation types during winter. Each phenology class is evenly represented, ranging from ephemeral/discontinuous snow covers to seasonal/continuous snow covers lasting > 6 months in most winters. The variability in seasonality is generally driven by steep elevation gradients with lower elevations classified as ephemeral or transitional and higher elevations as seasonal snow
	Transitional	0.33 M·km^2^ (< 1%)	0.24 M·km^2^ (< 1%)	0.09 M·km^2^ (6%)	
	Seasonal	0.23 M·km^2^ (< 1%)	0.19 M·km^2^ (< 1%)	0.03 M·km^2^ (2%)	
Montane forest	Ephemeral	0.76 M·km^2^ (1%)	0.75 M· km^2^ (1%)	0.02 M·km^2^ (1%)	Snow depth tends to be moderate to deep, ranging from 0.5—3 m, and snow density increases throughout the winter season. Characterized by mixed forests, with forest types and seasonal meteorology controlling densification rates. Phenology is most commonly transitional, in which temperatures remain cold enough to support snowpack development and sustained snow cover for 2—4 months, but ephemeral characteristics are common in shoulder seasons (e.g., late fall and early spring)
	Transitional	4.67 M·km^2^ (9%)	4.55 M·km^2^ (9%)	0.02 M·km^2^ (1%)	
	Seasonal	0.59 M·km^2^ (1%)	0.59 M·km^2^ (1%)	< 0.01 M·km^2^ (< 1%)	
Boreal forest	Transitional	3.78 M·km^2^ (7%)	3.78 M·km^2^ (7%)	< 0.01 M·km^2^ (< 1%)	Snow is shallow to moderate in depth, 0.3—1.5 m, and density is the lowest due to cold temperatures and low exposure to wind. Dominated by coniferous forest. Seasonal snow is most common, in which snow cover is sustained during winter in most years, usually lasting for > 6 months. Transitional areas occur along the lower latitude boundary of the boreal forest, where snow cover usually persists continuously for 3–6 months but ephemeral characteristics may present during shoulder seasons
	Seasonal	8.38 M·km^2^ (15%)	8.38 M·km^2^ (16%)	< 0.01 M·km^2^ (< 1%)	
Tundra	Ephemeral	2.06 M·km^2^ (4%)	2.02 M·km^2^ (4%)	0.04 M·km^2^ (3%)	Snow is generally shallow due to limited precipitation, though snow depths can exceed 5 m in drifts. Density is high in areas compacted by wind, but low in less wind exposed areas. Very cold with little to no tree cover. Snow cover phenology is most pronounced in this class. Ephemeral snow with short durations of snow cover < 2 weeks are common in cold deserts, however snow can occur in all seasons. Seasonal/continuous snow cover is the most common tundra seasonality class, with many areas regularly exceeding 7 months of sustained snow cover
	Transitional	1.80 M·km^2^ (3%)	1.77 M·km^2^ (3%)	0.03 M·km^2^ (2%)	
	Seasonal	8.96 M·km^2^ (17%)	8.94 M·km^2^ (17%)	0.02 M·km^2^ (2%)	
Ice	Perennial	14.47 M·km^2^	2.20 M·km^2^	12.27 M·km^2^	Permanent snow and ice cover
	TOTAL	144.7 M·km^2^	98.1 M·km^2^	46.6 M·km^2^	

Figure 2c–f highlight the differences between the traditional meteorological snow classes (panels c, e) and the combined SnowMAP classes (panels d, f) produced by adding snow phenology. This addition, shown by dark shades and hatching (seasonal) or lighter shades (ephemeral), distinguishes differences in snowpack characteristics. For example, Washington State’s Cascade Range (Fig. 2d,f) has persistent seasonal snowpacks at higher elevations and encompasses portions of maritime, tundra, boreal forest, and montane forest meteorological snow classes. These areas have longer durations of snow cover and are expected to have deeper snowpacks relative to the surrounding areas. At lower elevations, there are short-lived ephemeral snowpacks, predominantly within the maritime and prairie snow classes. These ephemeral snowpacks are highly sensitive to small seasonal variations in temperature and precipitation and tend to extend further on exposed south-facing slopes than on north-facing slopes (see Fig. 2e,f, circled area). Snow cover records indicate more transitional snow areas with increased snow persistence in river valleys on the eastern side of the Cascade Range and north-facing slopes (see Fig. 2f, dark yellow shades), which were classified as ephemeral in the meteorological classification.

### SnowMAP class meteorological, snow cover, and snow depth characteristics

**Fig. 3:**
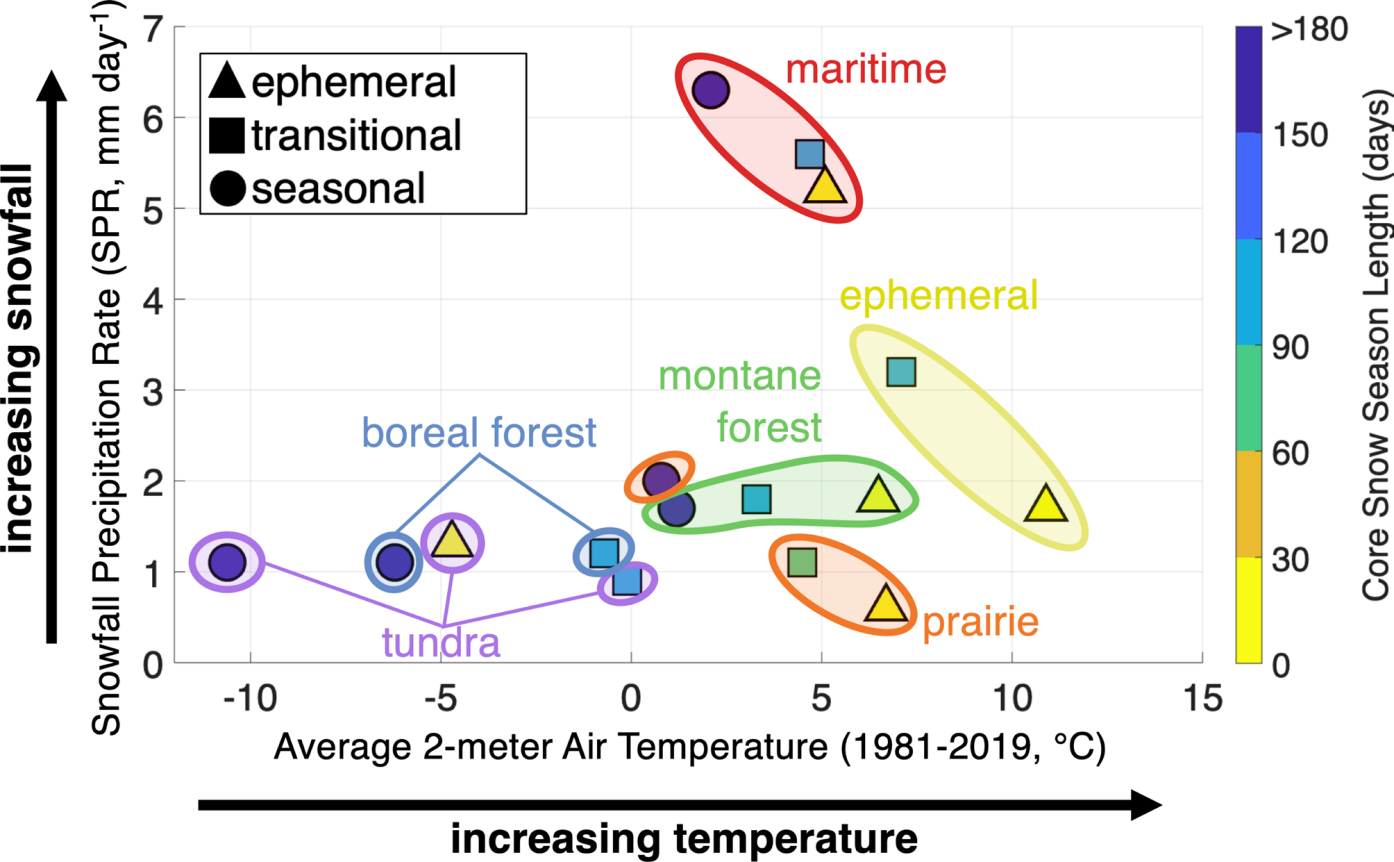
Snow classes have distinct snow cover, precipitation, and temperature characteristics. Climatological summary of combined SnowMAP classes (excludes no snow and permanent snow/ice). The plot includes snowfall precipitation rate (SPR, 1981–2019 via Sturm and Liston 2021), average 2-meter air temperature from 1981–2019 (ERA5-Land), and snow cover metrics (2000–2023 via Johnston et al., 2023). The median of each metric, considering all pixels within each SnowMAP class, is shown. The marker shape indicates the seasonality class (triangle = ephemeral, square = transitional, and circle = seasonal), and color-coded bounding shapes show the meteorological snow classes. Marker color indicates the median length of the longest continuous snow-covered period (i.e., core snow season) in days. All summary statistics are included in Supplementary Table 2.

SnowMAP classes have distinct snow cover, precipitation, and temperature characteristics (Fig. 3). Across the meteorological ephemeral, prairie, and maritime snow classes, increases in snow phenology classes from ephemeral (triangles) to seasonal (circles) are associated with increases in snowfall, decreases in temperature, and a longer core snow season (i.e., the longest sustained period of snow cover). For the ephemeral, prairie, and maritime snow classes, snowfall rates differ among the phenology classes, but there is no notable relationship between snowfall rates and phenology for the montane forest, boreal forest, and tundra snow classes. For example, in the ephemeral, prairie, and maritime classes, median snowfall rates in transitional classes are 0.4 to 1.5 mm/day higher than those with ephemeral phenology. For prairie and maritime, seasonal snowfall rates are 0.7 to 0.9 mm/day higher than those with transitional phenology.

For ephemeral, prairie, montane forest, and maritime meteorological snow classes, which are typically found in lower latitudes, modest changes in air temperature change the phenology classes, as compared to the very cold tundra and boreal forest classes, whose transitional and seasonal snow areas are less sensitive to temperature. In boreal forests and tundra, transitional classes have long-term (1981–2019) average temperatures near 0 °C, whereas seasonal snow classes have temperatures below − 5 °C. This temperature difference results in longer median core snow seasons (> 6 months) in these seasonal snow classes as compared to transitional areas (3 to 4 months). Uniquely, ephemeral tundra, which has long-term average temperatures around − 5 °C, experiences highly intermittent snow cover and is colder than transitional tundra (approximately 0 °C). Ephemeral tundra is primarily located on the Tibetan Plateau, a high-elevation, arid, and wind-exposed area known for systematic wet biases in gridded precipitation products^45^. Cold temperatures, high wind speeds, and dry conditions may also lead to high sublimation rates, which explains the highly ephemeral and shallow snow cover in the region. The median length of the core snow season increases from approximately 1 to 2 weeks for all ephemeral phenology regions to 2 to 4 months in transitional regions, and then to more than 6 months in seasonal snow areas. Additionally, while transitional and seasonal snow classes tend to have persistent snow cover for more than 80% of the time between the first and last date with observed snow cover (i.e., snow season persistence), this value is most commonly between 25% and 60% in ephemeral snow classes (see Supplementary Table 2).

**Fig. 4:**
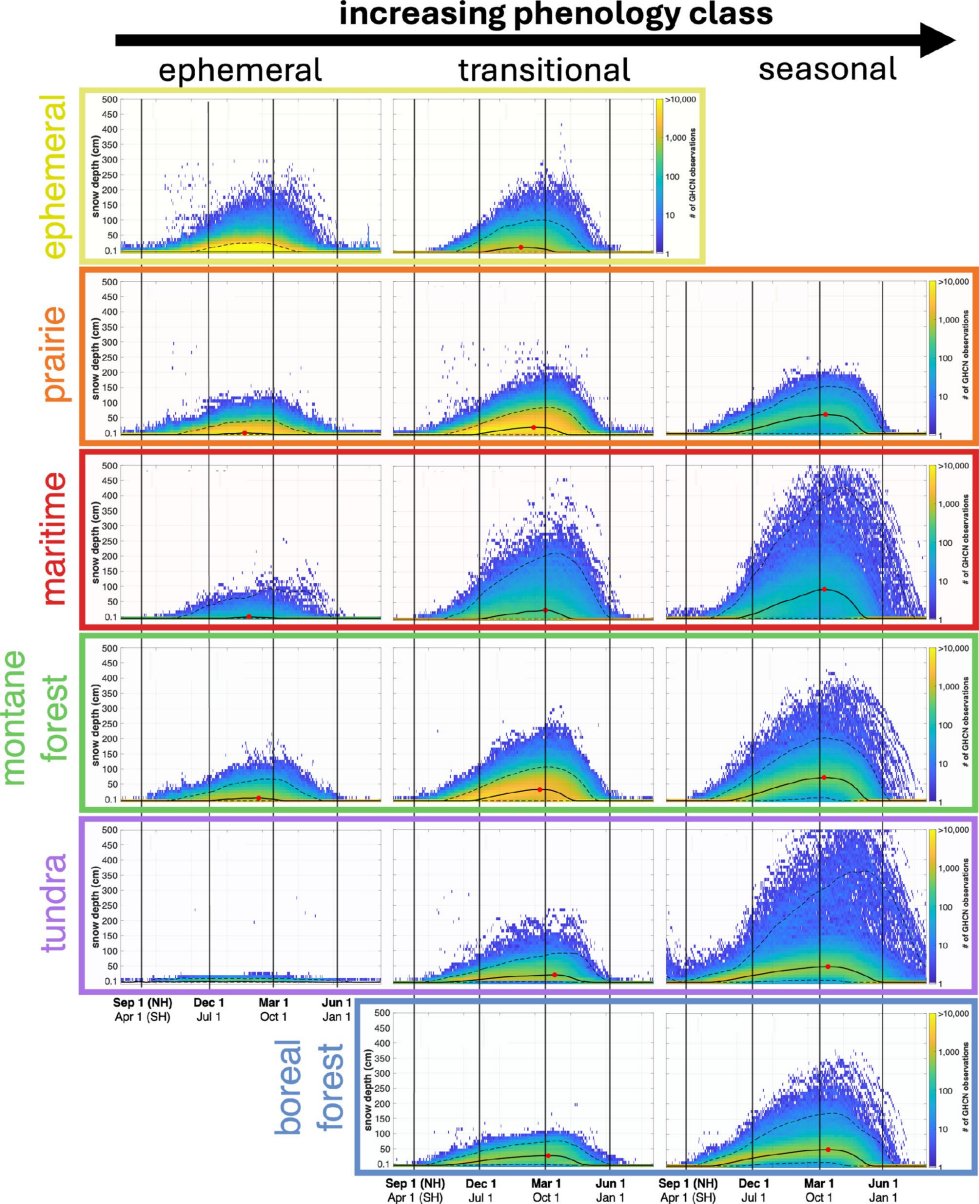
Increasing snow phenology classes correspond to deeper and more variable snow depths, but less variable snow cover conditions and later melt-out. Summary of Global Historical Climatology Network (GHCN) daily snow depth observations from 1981–2025 by SnowMAP class. Within each plot, colors indicate the density of observations (high = yellow, low = blue). Seasonal timing (Northern Hemisphere, NH; Southern Hemisphere, SH) is indicated by vertical lines. Columns from left to right represent ephemeral, transitional, and seasonal snow phenology classes, while rows correspond to meteorological snow classes (Sturm and Liston 2021). Solid black curves indicate the median depth considering all observations for the specified date. The peak median snow depth, when non-zero, is indicated by a red dot. Dashed lines bound the 95% interval of observations (2.5th – 97.5th percentile). Corresponding statistics are included in Supplementary Table 3.

Long-term, daily snow depth records from the Global Historical Climatology Network (GHCN) indicate that observed snow depths and peak snow depth magnitudes increase, and the peak snow depth shifts later in the season as snow phenology classes transition from ephemeral to seasonal (left to right, Fig. 4). The GHCN record confirms that transitions from ephemeral to seasonal, which are indicative of increasingly stable snow cover, also correspond to a larger range of snow depths. The GHCN records reveal that a distinct evolution of snow depth exists within each meteorological snow class.

Due to the large proportion of observation sites and SnowMAP class extents located within the Northern Hemisphere, the peak depth timing analysis was only conducted for the Northern Hemisphere. In the Northern Hemisphere’s ephemeral snow regions, the timing of the seasonal snow depth peak is inconsistent, and snow depth tends to be very shallow (median peak < 10 cm). In many cases, snow may not be present at all, as snow depths of zero are commonly observed during all seasonal periods. Snow depths approach 30 cm in the snowiest years and locations (i.e., ‘upper bound’ or 97.5th percentile, dashed line) and, in rare cases, depths exceeding 1 m have been observed (left column, Fig. 4). In the snowiest years, peak snow depths in ephemeral snow classes occur in mid-February (February 11–21), with the exception of ephemeral tundra, which lacks a distinct peak.

Transitional classes in the Northern Hemisphere have snow depth peaks that typically occur around March 1, with deeper snow (median peak of 15–30 cm) compared to ephemeral phenology classes (middle column, Fig. 4). The timing of the median peak snow depth ranges widely from January 27 (transitional ephemeral) to March 15 (transitional tundra), with typical peak snow depths between 15 cm and 36 cm. In the snowiest seasons (97.5th percentile), peak snow depths occur 2 to 4 weeks later in the season and depths range from 80 cm (boreal forest) to 213 cm (maritime). Even in snowy seasons, snow depths exceeding 1 m are rare in transitional tundra and boreal forests, whereas other transitional classes show peaks approaching 2 m (transitional ephemeral, prairie, and montane forest) or exceeding 2 m (transitional maritime). In normal (i.e., median) years, snow depths in transitional classes tend to be moderate, approaching peaks of 50 cm. However, variability remains high, with shallow (< 20 cm) or no snow (0 cm) observations in all periods, including mid-winter.

Seasonal snow classes in the Northern Hemisphere have distinct snow depth evolutions, with median peaks in all classes occurring after March 1 and with the deepest median depths (51–93 cm) of all phenology classes (right column, Fig. 4). The timing of the median peak depth is similar across all seasonal snow classes, ranging from March 10 to 16, though in the snowiest years (97.5th percentile), peak snow depths may occur much later, and can be as late as mid-May in seasonal tundra. The deepest observed snow depths also occur in seasonal snow classes, with peak depths in the snowiest years ranging from 154 cm (seasonal prairie) to more than 400 cm (seasonal maritime). In seasonal montane and boreal forests, the maximum observed depths are around 3.5 m, while maximum depths in seasonal maritime and tundra classes exceed 5 m. In contrast to ephemeral and transitional snow classes, the densest area of snow depth observations for seasonal classes (right column, Fig. 4) is shifted towards deeper snow depths (December 1 to March 1) such that even in the least snowy seasons (the ‘lower bound’, i.e., 2.5th percentile), many seasonal classes maintain a continuous snowpack.

### Drivers and context of SnowMAP classes: elevation, latitude, land cover, people, and infrastructure

**Fig. 5:**
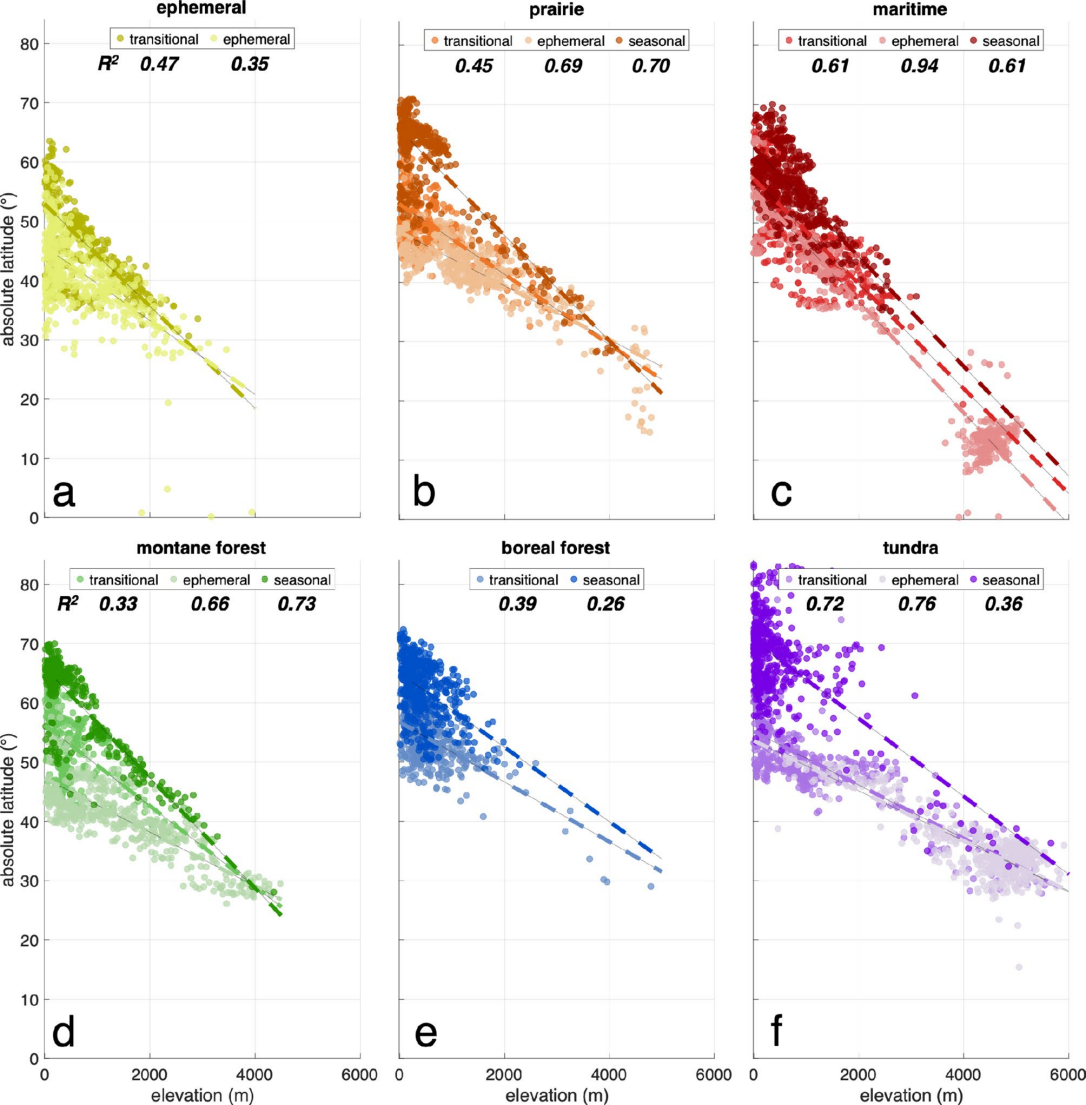
Elevation and latitude have a significant influence on snow phenology within each meteorological snow class. Scatter plots of elevation (x-axis) compared to latitude (y-axis) for each meteorological and phenology SnowMAP class. Each panel shows the specified meteorological snow class, with darker colors indicating an increasing snowpack phenology class. Latitudes are converted to absolute values before plotting. To improve visualization, plots were generated using 500 random samples extracted from each class globally, while regression fitting was performed using all data for each class.

To better understand the drivers and underlying characteristics of the SnowMAP classification, we summarize relationships between snow phenology classes and elevation, latitude, land cover, and land use (via population and infrastructure metrics). As expected, elevation and latitude strongly influence snow seasonality within each meteorological snow class (Fig. 5). Seasonal snow is more prevalent at higher latitudes (y-intercept = 62.8° to 70.5°). However, a negative linear relationship (R^2^ = 0.33 to 0.94) exists between elevations (increasing) and the latitude (decreasing) at which a given snow class can develop (see Supplementary Table 4). Within 40 degrees of the equator, snow is overwhelmingly ephemeral in nature, even at high elevations (see ephemeral tundra, Fig. 5f). Low-latitude ephemeral snow within 20 degrees of the equator is mostly located in the Andes coastal mountain range of South America (see ephemeral maritime, Fig. 5c). These results confirm that elevation and latitude shape distinct snow climates and their phenology through their controls on air temperature and precipitation.

**Fig. 6:**
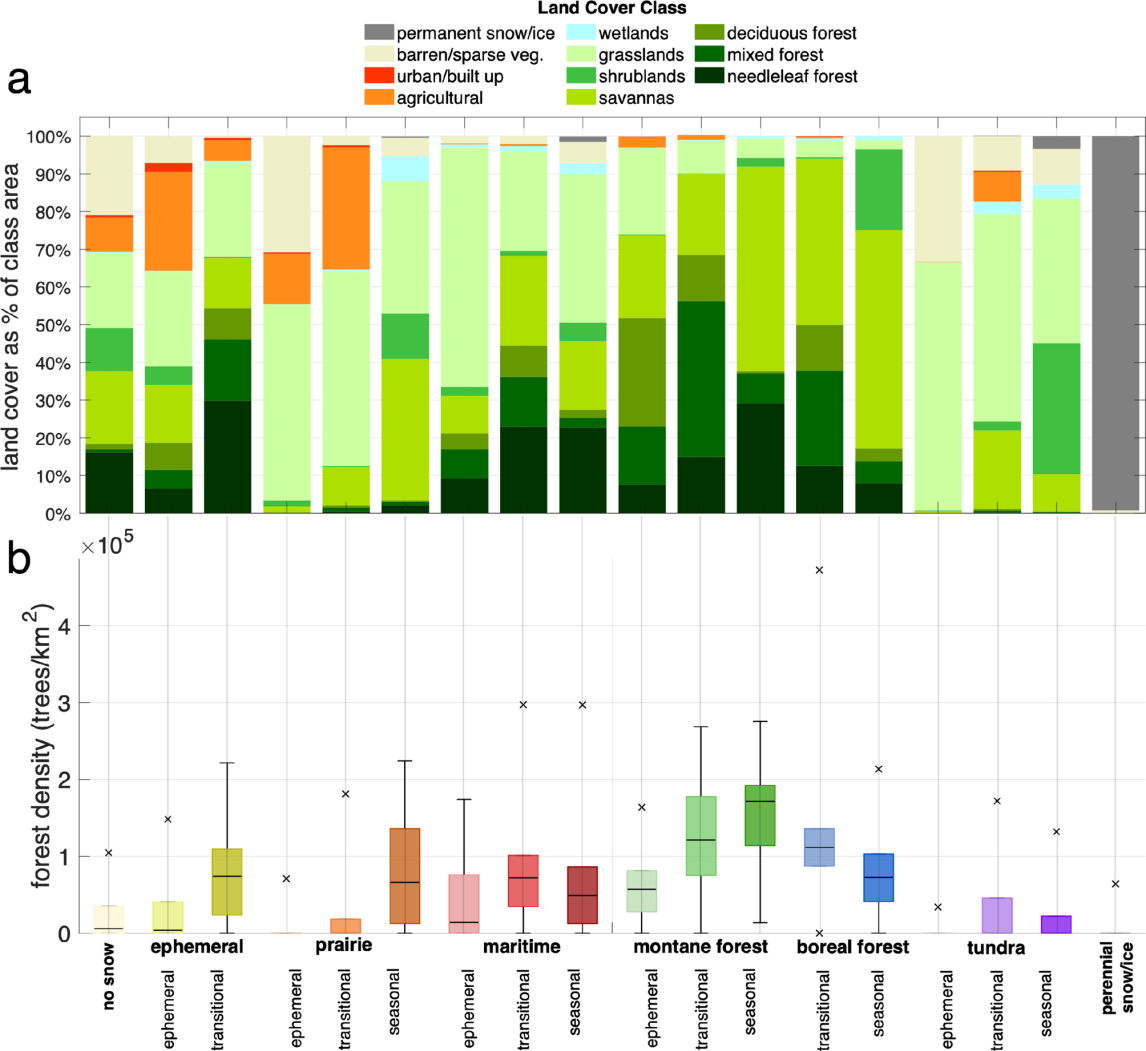
A diverse range of land cover and vegetation conditions exists within each SnowMAP class. For each SnowMAP class, (**a**) the percentage of class covered by the specified land cover class (see Supplementary Table 5 for corresponding statistics) and (**b**) the distribution of forest density in trees per km^2^ are shown. For (b), meteorological snow classes are bolded on the x-axis, and high and low extreme values are the 1st and 99th percentiles, while each box includes the 25th, 50th, and 75th percentiles of forest density values.

A diverse mosaic of land cover and land use classes exists in each SnowMAP class (Fig. 6, Supplementary Table 5). Regardless of the meteorological snow class name, a range of vegetation conditions (e.g., vegetation type, density) is evident, which varies across phenology sub-classes. For example, while all maritime, montane forest, and boreal forest classes have a large proportion of forests (17–68%), non-forested dominant areas (i.e., savannas, shrublands, grasslands, barren/sparse) are even more prevalent in many classes (30–82%). Tundra and prairie snow classes primarily consist of non-forested areas and barren/sparse vegetative cover (64–100%); however, shrublands and dense woody forests (> 100,000 trees/km²) are also present (Fig. 6a,b). Due to its broad spatial extent, the meteorological ephemeral class also encompasses a diverse range of land cover types, including all forest types, a variety of forest densities (up to 220,000 trees/km^2^), as well as significant areas with non-forest vegetation and croplands. Similarly, maritime snow areas are comprised of mixed forests of varied densities (up to 300,000 trees/km^2^), as well as open areas with shorter vegetation. Local climates and associated snow phenology have clear implications for the dominant properties of the forests and land cover, as well as the mechanisms driving snow’s spatial variability and human interactions (i.e., land use) in regions with different snowpacks. In turn, these land cover types strongly influence local-scale snow variability by controlling processes such as wind drifting, snow canopy interception, and shading.

**Fig. 7:**
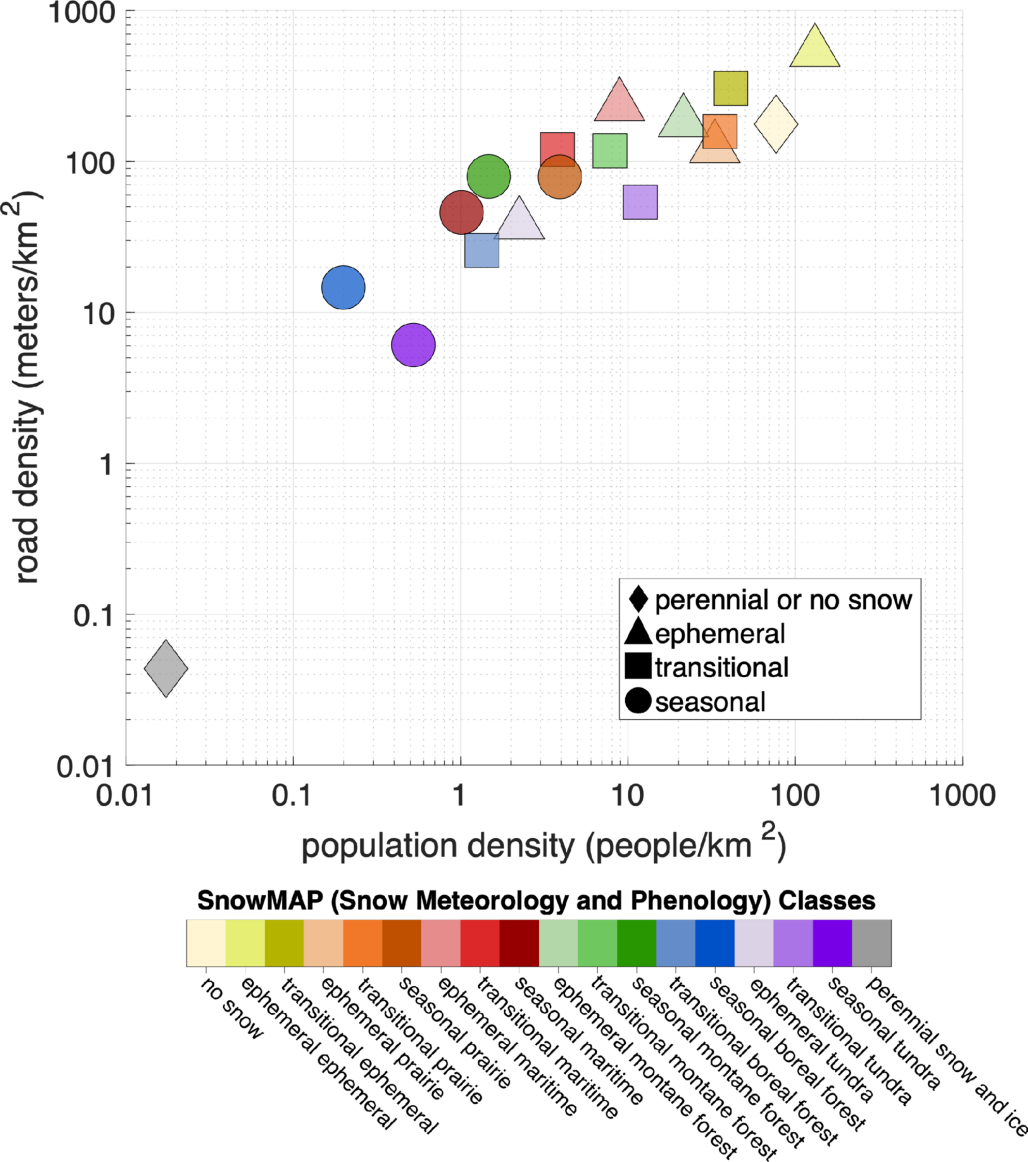
Snow climates influence where we live and travel, with most development occurring in the no snow, ephemeral, or transitional snow classes. Scatter plot of population density (via Worldpop 2020) compared to road density (GRIP4, all roadway types) for SnowMAP classes. The marker shape corresponds to the snow seasonality class, with color corresponding to the SnowMAP class shown in the legend (bottom).

Ephemeral and transitional snow climates host higher population densities and more transportation infrastructure than seasonal snow regions (Fig. 7). Despite urban areas’ limited extent – less than 2.5% of each snow class area (Fig. 6a) – snow imposes an outsized impact on the movement of goods and people in more populous regions. A distinct power-law relationship exists between the density of people living in a given snow class (increasing) and the density of the transportation network (increasing). The densest road networks (530 m of roadway per km^2^) are found in areas where both the meteorological and snow phenology classes are ephemeral (‘ephemeral-ephemeral’). Of the approximately 8 billion people on Earth as of 2020^45,46^, 93.5% of the global population live in either the no snow (73.4%) or the ephemeral-ephemeral SnowMAP classes (20.1%) (see Supplementary Table 6). Of the remaining 6.5%, only ephemeral and transitional prairie contains more than 1% of the global population. Between 0.1% and 1% of the population lives in the ephemeral montane forest, transitional montane forest, transitional tundra, or transitional ephemeral snow class, while the proportion of the global population included in each of the 10 (of 18) remaining SnowMAP classes is less than 0.1%.

### SnowMAP in practice: winter recreation and hydrologic benchmarks

SnowMAP classes have value beyond the cryosphere research community, as demonstrated by the extensive literature that already uses existing snow classification schemes^26,35,37–44^. Here, we present two case studies that highlight how SnowMAP can provide practical insights. The first case study explores ski areas and how SnowMAP classes can characterize their snow conditions, offering a potential resource for the winter recreation industry. The second case study examines changes in runoff seasonality between adjacent watersheds with different dominant SnowMAP phenological classes.

#### Assessing ski area snow conditions and characteristics

**Fig. 8:**
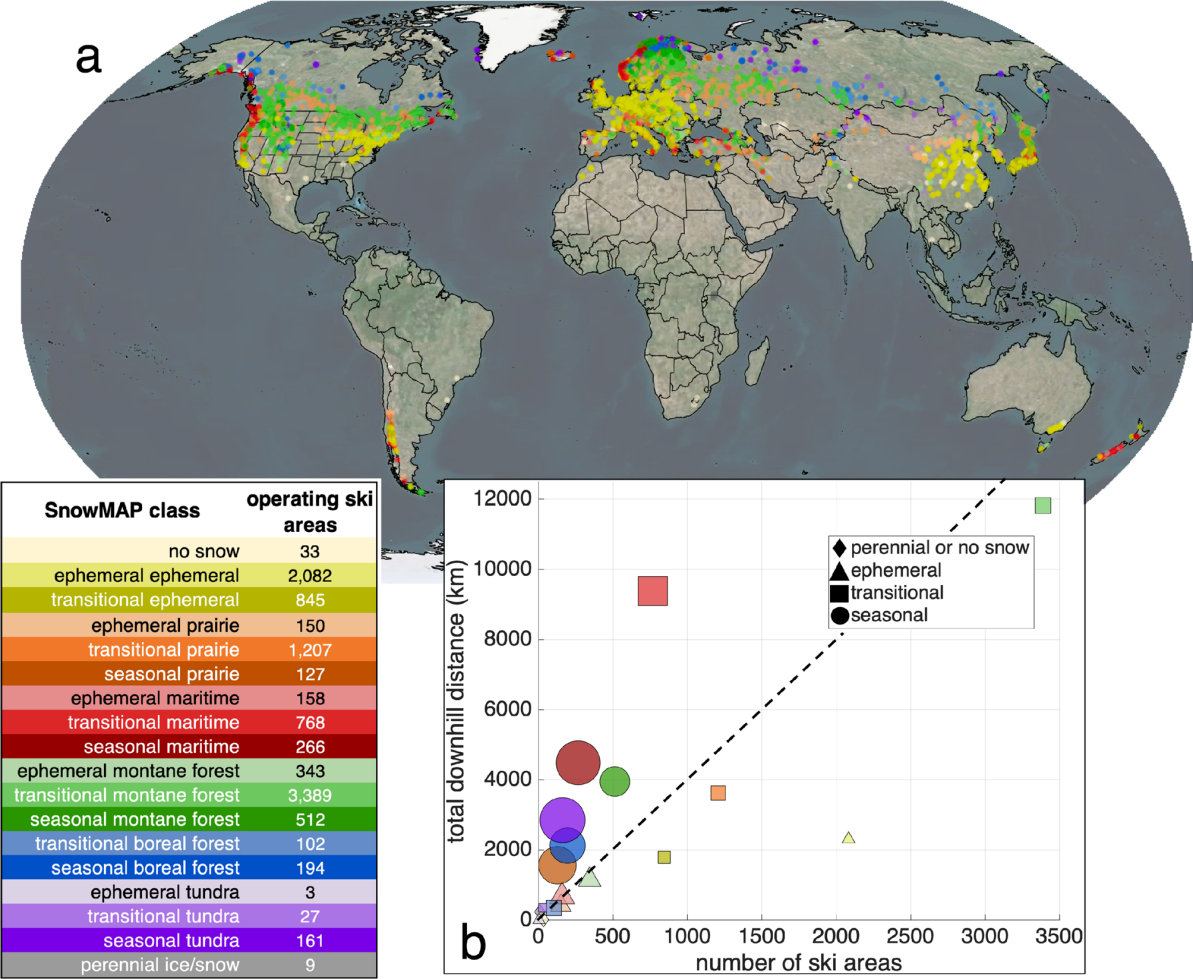
Snow classes can be used to characterize global ski areas and their vulnerability to a changing climate. (**a**) Global map of ski areas (via OpenSkiMap.org, as of April 2025) colored by SnowMAP class (*N* = 10,376). Class colors correspond to those shown in the inset table, which also includes the total number of ski areas within each class. (**b**) Relationship between the number of ski areas (x-axis) and total downhill distance (y-axis) within each snow class for global ski areas (via OpenSkiMap.org, as of April 2025). Marker size is proportional to the ratio of total downhill distance divided by the number of ski areas.

Millions of people around the world enjoy recreational activities in the wintertime, with skiing and snowboarding being among the most popular^47^. Characterizing the global distribution of ski areas using snow classes allows inferences to be made about the nature of the snowpacks at these locations (e.g., deep, cold, persistent snow versus marginal, warmer, wet snowpacks), the prevalence of each type of ski area, and potentially their vulnerability to shifts in regional weather and climate conditions. The SnowMAP class for each of the more than 10,000 operational ski areas globally^48^ was summarized by location (Fig. 8a) and by number and size (Fig. 8b). For phenology classes, ski areas were most prevalent in the transitional snow sub-class (61% of all ski areas) and rarest in seasonal areas (12%). Ski areas are also surprisingly prevalent in ephemeral snow areas (27%), even though snow cover is already intermittent in these regions and likely to become more so^33^. The high density of snow classes within ephemeral snow areas likely reflects the relatively high populations in these regions, as can be seen by the large number of ski areas in the eastern United States, Europe, and eastern China. Ski areas are found in all 18 SnowMAP classes but are most common in the transitional montane forest (3,389 ski areas; 33% of all global ski areas), ephemeral ephemeral (2,082; 20%), and transitional prairie (1,207, 12%) classes. These three snow classes encompass 65% of all global ski areas (class properties are detailed in Figs. 1, 3 and 4, and in Table 1). Larger resorts with more skiable distance (12.2 km per ski area) are primarily located in transitional maritime areas. The many ski areas located in transitional montane forest and ephemeral ephemeral snow classes (5,471; 53%) are generally smaller (3.5 km and 1.1 km per ski area, respectively). Figure 8b highlights this relationship between the downhill distance and the number of ski areas. Most ephemeral and transitional snow classes are located on or below the bisecting dashed line (4 km per ski area), in contrast to seasonal snow classes (circles), which lie above this line. The trade-off between ideal snow conditions and skiable terrain, which are regularly present in mountainous areas (commonly seasonal snow), and the proximity to large population centers with smaller elevation gradients (commonly ephemeral snow) likely drive these dynamics. The transitional ephemeral class, which covers less than 1% of global land areas (Table 1), contains a relatively high number of ski areas (845; 8%). The prevalence of ski areas in these regions suggests that the locally persistent or deeper snows are suitable for winter recreation.

#### Linking snow phenology and basin hydrology

Snowpack properties like water-holding capacity, ripeness, and SWE, as well as snow cover observations of accumulation and melt timing (i.e., phenology), provide insights into regional hydrologic processes. Notably, understanding differences in snow phenology can play a critical role in identifying seasonal water availability and hydrologic hazards, such as floods and droughts. This information is critical for forecasting and risk assessment, particularly in cold regions.

**Fig. 9:**
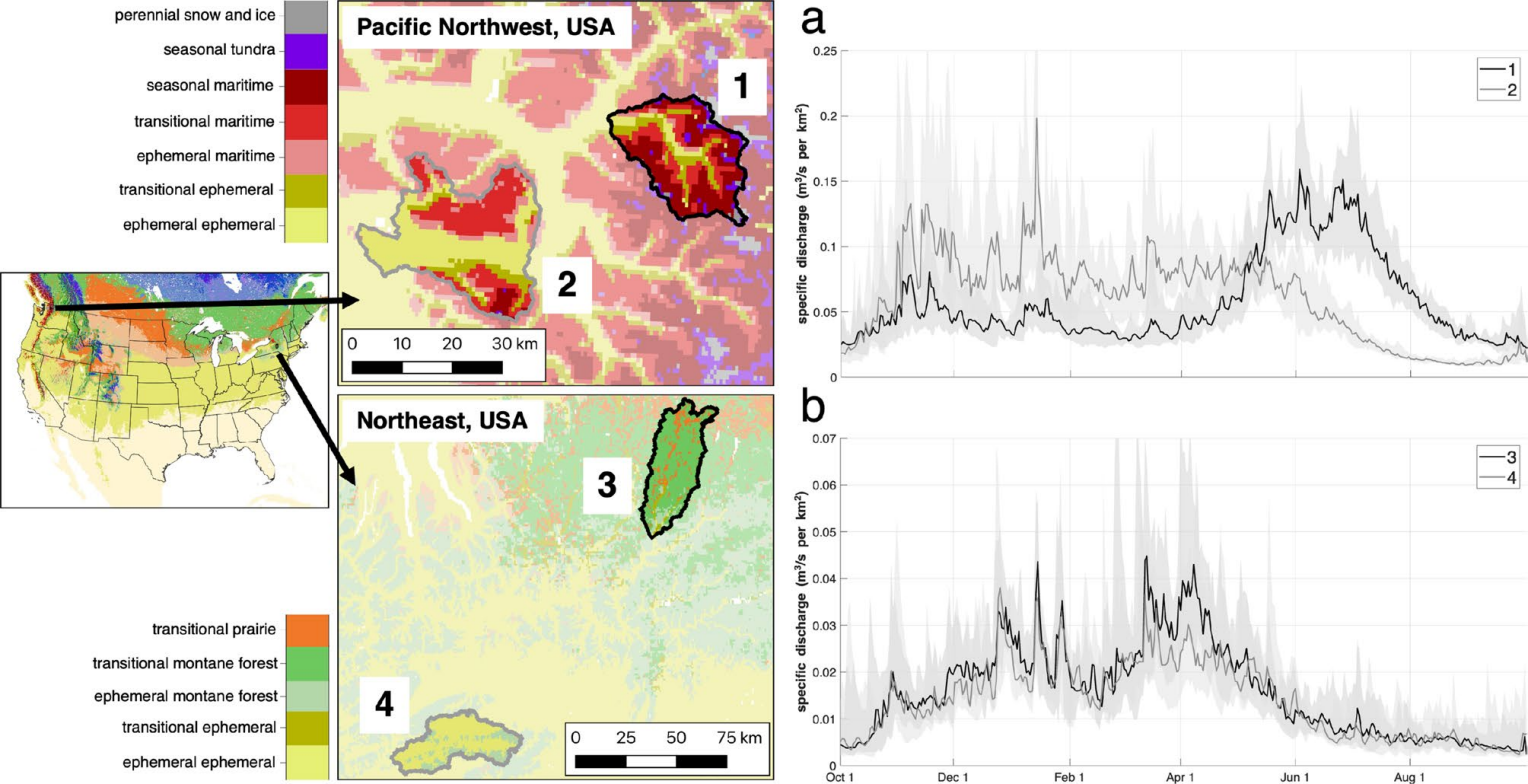
Adjacent basins with different dominant SnowMAP phenology classes have distinct runoff seasonality. SnowMAP classes are shown for the United States (left) with arrows indicating the locations of the zoomed-in basin pairs within the Pacific Northwest, USA (top), and the Northeast, USA (bottom). Each basin was identified using the GAGES-II reference watershed dataset (Falcone et al., 2010). Streamflow data for each basin from the United States Geological Survey (USGS) is summarized daily for the (**a**) Pacific Northwest (2006–2023) and (**b**) Northeast (2007–2023) basin pairs using the median (solid lines) and quartiles (shaded areas) for the indicated periods. Note that the lighter shading corresponds to the lighter lines.

The GAGES-II^49^ hydrologic data archive includes thousands of gaged reference basins (i.e., watersheds with minimal human modification) across the United States, as well as detailed basin characteristics (e.g., land cover and land use, topography). To evaluate the value of SnowMAP for identifying basins with unique streamflow seasonality, we selected two sets of adjacent, similarly sized GAGES-II watersheds in the eastern and western United States with comparable land cover but differing snow phenology (Table 2). By identifying proximal basins, we aim to limit the influence of mesoscale (< 500 km) precipitation variability. These basins are shown in Fig. 9 and include one pair in the Pacific Northwest (western USA), characterized by mountainous terrain, and a second in the Northeast (eastern USA), characterized by gentle slopes. Streamflow climatology was produced using periods of overlapping streamflow observations for water years across both paired basins from 2000 (start of the MODIS record) to 2023 (the last year included in the snow phenology dataset^33^.

**Table 2:** Basin characteristics. Including descriptions of each USGS gaged basin within the paired basin comparison.

Basin ID	USGS gage	Gage		Name	Area	Mean elevation	Snow phenology class			Basin description
		Latitude	Longitude				Ephemeral	Transitional	Seasonal	
1	12,182,500	48.526	-121.4154	CASCADE RIVER AT MARBLEMOUNT, WA	444 km^2^	1260 m	11%	37%	53%	mostly evergreen forest, some shrublands and barren land, small glaciated area (~ 1 km^2^) at headwaters, undeveloped; 56% slope; northwest facing; meteorological snow classes are 68% maritime, 21% ephemeral, 9% tundra, 3% ice
2	12,167,000	48.261	-122.048	NF STILLAGUAMISH RIVER NEAR ARLINGTON, WA	684 km^2^	655 m	54%	43%	4%	mostly evergreen and mixed forests, some shrublands, minimal development; 33% slope; southwest facing; meteorological snow classes are 50% maritime and 50% ephemeral
3	01502500	42.378	-75.406	UNADILLA RIVER AT ROCKDALE NY	1346 km^2^	452 m	7%	93%	0%	mixed forests and agricultural lands, minimal development; 9% slope, south facing; meteorological snow classes are 82% montane forest, 14% prairie, 4% ephemeral
4	01552000	41.325	-76.912	LOYALSOCK CREEK AT LOYALSOCKVILLE, PA	1130 km^2^	509 m	100%	0%	0%	mostly mixed forests, partially agricultural lands, minimal development; 13% slope; south facing; meteorological snow classes are 69% ephemeral, 31% montane forest, 1% prairie

Using this approach, SnowMAP identified strikingly different runoff seasonality between the pair of Pacific Northwest basins (Fig. 9a), in which one was dominated by transitional and seasonal snow phenology and sits at a higher elevation (basin 1), and another by ephemeral and transitional snow phenology (basin 2, Table 2). In basin 1, there is an extended low flow period during the cold season (December – April), followed by a distinct runoff peak in late spring and summer (May – August). This contrasts with basin 2, which lacks a distinct seasonal runoff peak and exhibits elevated flows from November through May. For the Northeast basins (Fig. 9b), streamflow seasonality was less distinct (basins 3 and 4). Basin 3 is dominated by transitional snow phenology (93% coverage) compared to basin 4, which falls 100% within the ephemeral phenology class (Table 2). However, while the streamflow seasonality remained similar from October through mid-March, specific discharge was elevated in basin 3 compared to that of basin 4 from mid-March into mid-April (+ 20% to + 70%). This coincides with the spring snowmelt period for the region, suggesting that snow phenology classes within SnowMAP can provide insights as to the regional hydrologic cycle.

Such regional evaluations of snow class distributions are recommended to ensure that study basins accurately reflect the key snow characteristics that influence regional hydrologic cycles. Still, there are nuances in how snow may influence seasonal runoff, likely depending on the distribution of snow classes within a given basin. For example, a watershed that lies mostly within the seasonal snow class is expected to exhibit a distinct late-spring increase in runoff, compared with those that exhibit greater variability in snow conditions (i.e., ephemeral and transitional snow classes). Further exploration of basin snow class phenology for individual winters may be critical for identifying basins with the least predictable streamflow seasonality, which we hypothesize are those that shift between phenological classes from year to year.

## Discussion

In this study, we describe and demonstrate the utility of a new global snow classification, termed SnowMAP (Snow Meteorology and Phenology), produced by merging existing long-term average meteorology-based snow classes^18,26^ and snow phenology classes produced from a multi-decadal record of snow cover^33^. SnowMAP, at its core, is a framework for unifying existing snow datasets and their descriptions. As such, any reuse of this dataset should also acknowledge the underlying data products^50,51^. The SnowMAP dataset is a tool for describing expected snow conditions and their seasonal evolution and is not intended to replace sub-seasonal physical modeling or observations. Conditions within each class are expected to vary annually, especially in winters with anomalous conditions (e.g., warm and dry, cold and wet), warranting further investigation into interannual variability and snow class uncertainty. European Centre for Medium-Range Weather Forecasts (ECMWF) Reanalysis, 5th Generation Land^52,53^ gridded reanalysis products are used by Sturm et al., (2021)^26^ to produce temperature records and identify areas of high and low precipitation. While global evaluation suggests ERA5 temperatures are generally accurate^54^, there are known precipitation biases, including a tendency to overestimate precipitation in dry regions and increased uncertainty in remote areas with sparse station coverage, including high-altitude regions^45,54^. The ERA5-Land downscaling process used by Sturm and Liston (2021)^26^, which uses MicroMet^55^ to downscale 0.1° x 0.1° gridded ERA5-Land data to 10 arc second resolution (~ 0.003°), relies on spatially and temporally constant lapse rates, which may introduce additional uncertainties. Snow-cover mapping using the Normalized Difference Snow Index (NDSI) also has limitations, particularly in areas of poor solar illumination, regularly cloud-occluded regions, and forested regions^33^. These data limitations propagate into the SnowMAP product and should be considered before its use.

Even so, in this study, we identify multiple phenological classes (ephemeral, transitional, and seasonal) that exist within each broader meteorological snow class group (Table 1), with ephemeral and transitional classes comprising a large portion of each meteorological snow class (ranging from 30% tundra to 98% prairie). Since ephemeral and transitional areas have unstable snowpacks and high inter-annual variability in snow cover conditions^33^, these areas are expected to take on properties of another class during regular transitional periods, like the ephemeral snow class (e.g., wet, shallow, discontinuous snow cover) rather than the snow properties described by Sturm and Liston (2021)^26^. In the ephemeral tundra snow class, snow may not be present at all in mid-winter. Within each meteorological class, we show that as snow phenology transitions from ephemeral to seasonal, the observed snow depths increase, and the peak snow depths shift to later in the season (Fig. 4). This is consistent with findings linking regionally deeper snow to longer snow cover durations^56^ and with physical laws governing snowmelt^57^.

Geographic variables, elevation and latitude, are shown to affect the development of phenology sub-classes within each meteorological snow class (Fig. 5), aligning with known controls of snow line elevations^58,59^. Land cover (Fig. 6) is highly variable within each SnowMAP class, even when a dominant cover type is apparent. At the sub SnowMAP pixel scale (< 1 km), vegetation will modulate local snow and ecological conditions (e.g., Komarov and Sturm 2023^60^). SnowMAP also reveals a link between snow phenology and built-up regions, showing much higher populations and roadway densities in ephemeral areas relative to other classes (Fig. 7).

As part of an international collaboration, *The International Classification for Seasonal Snow on the Ground*, Fierz et al., (2009)^61^ standardized snow characterization and measurement approaches, by providing guidance on classifying snow grain shape, size, as well as for measuring snow depth, density, hardness, liquid water content, temperature, impurities, and layer thickness, among other characteristics. While this document is invaluable for detailing measurement techniques and provides a glossary of key snow terminologies and symbols, it does not seek to map snow characteristics geographically. Like SnowMAP, Royer et al. (2021)^62^ mapped snow classes using in situ observations along a latitudinal transect (47 °N to 83 °N), which spans a distinct gradient in temperature and precipitation in Eastern Canada. While like the Sturm et al. (1995)^18^ approach, their method incorporates more detailed information on vegetation structure (e.g., shrub, open, or closed forest) and identifies a new class, termed polar desert above 74 °N, characterized by very dense, shallow snow layers. In the future, these and similar systems should be integrated with SnowMAP to provide improved snow class definitions and to identify relevant sub-classes not currently captured by SnowMAP.

The potential advantages of the SnowMAP classification framework are extensive. In addition to using SnowMAP to link the global distribution of snow and ski areas (Fig. 8) and to explore the influence (or lack thereof) of snow on watershed-scale runoff seasonality (Fig. 9), there are numerous other research opportunities that could benefit from incorporating snow phenology. For example, snow classes may be related to critical areas for water supply and used for siting weather stations (e.g., Raleigh et al., 2025^63^), to forecast/hindcast snow climates (e.g., Nolin et al., 2005^64^), as predictors in machine learning models (e.g., Dunmire et al., 2024^65^), to explore engineering applications (e.g., snow loading, roadway trafficability), for field planning (e.g., identifying winter study sites), or to interpret remotely sensed snow signals (e.g., mixed signals in areas with multiple snow climates), among others. Another advantage is the dynamic representation of snow, which is not available from meteorological classes alone. For example, the snow phenology within SnowMAP provides insight into the uncertainty of snow properties. As a rule of thumb, ephemeral implies low confidence that snow is on the ground at any point in winter, transitional implies high confidence in snow presence in mid-winter but low confidence in the shoulder seasons, while seasonal implies high confidence in snowpack presence throughout the entire winter.

SnowMAP provides a practical, decision-ready framework for scientists across hydrology, ecology, and climate, as well as practitioners and communities seeking to better understand local snow regimes. Users can explore whether their system’s behavior (e.g., hydrologic, ecologic, economic) differs by SnowMAP class and use these classifications as a framework for generating physical insights into why those differences occur. Broader use will also support continued refinement of the classification and enable improved descriptions of snow property evolution. In the future, SnowMAP can be reissued on a decadal basis – or even annually – to track shifts in accumulation, melt, and persistence, providing representative maps of changing snow conditions over time. In summary, SnowMAP advances snow classification by linking seasonal patterns of accumulation and melt with snow’s physical properties. In doing so, it translates the complexity of snow into actionable global insights relevant to water security, ecosystems, infrastructure, and our personal winter experiences.

## Methods

### Producing the SnowMAP snow classification

The following steps are used to merge the seven snow meteorological classes^26^ and five snow phenology classes^33^ to produce the 18-class Snow Meteorology and Phenology (SnowMAP) snow classification dataset. The combination of these datasets relies on three inputs detailed in the ‘Data’ section below, including (1) the global (GL) 30 arc-second (1/120°) snow classification^50^, (2) the 0.01° snow seasonality classification^51^, and (3) the 30 arc-second GMTED2010 mean elevation dataset^66^. The workflow detailed below is documented and applied using a MATLAB function available at https://github.com/jjohns60/SnowMAP.

*Grid matching*: The snow seasonality class climatology (referred to as snow phenology classes in this work) is resampled to a 30 arc-second grid using nearest neighbor interpolation.

*Class combination*: Since the snow cover classification is applied on an annualized basis, and climatology snow cover classes for all years in the period (2000 to 2023) were produced by taking the average class rounded to the nearest whole number (i.e., 0 = no snow, 1 = ephemeral, 2 = transitional, 3 = seasonal, 4 = perennial). These values were combined with the 7 meteorological snow classes to produce any of 35 possible unique numeric codes.

*Class aggregation*: Classes not occurring in nature (e.g., no snow + permanent ice/snow) and those covering very small spatial extents (< 2% of the overlying meteorological class) were relabeled to the next most logical class. For example, areas classified as perennial boreal forest covered approximately 300 km^2^ globally and were merged with the seasonal boreal forest (> 8 million km^2^). In areas in which the meteorological class was ephemeral and the phenology class was no snow, a new ‘no snow’ class was introduced. In other cases, the proposed combination defaults to the meteorological snow classes (e.g., areas defined as permanent snow/ice in meteorological classes were retained), under the assumption that there are imperfections in MODIS-based SCA estimates due to cloud cover, vegetation effects, and surface features (e.g., sand flats) that can influence snow cover mapping accuracy. This aggregation step resulted in the identification of 18 regularly occurring snow classes, each covering more than 190,000 km^2^ globally.

*Snow cover artifact filtering*: In many no or low snow regions, small lakes and salt flats have similar optical properties to snowpacks, resulting in regular false snow classifications in the snow cover climatology. To identify and remove these areas, small, connected areas within broader no snow or ephemeral snow areas were automatically delineated and then dilated. In cases where the elevation of the dilated boundary was higher than that of the interior areas, and at least 90% of the pixels in the dilated region were either water or a lower-snow class than the interior area (e.g., the interior area was seasonal but all areas in the surrounding boundary were no snow), the interior area was set to match the dominant class in the dilated area. This greatly reduced the number of snow cover classification artifacts around water bodies.

### Data

#### Snow classifications

The global 30 arc-second (~ 1 km) seasonal snow classification^50^ and 0.01° (~ 1 km) snow seasonality climatology^51^ datasets, both available at the National Snow and Ice Data Center (NSIDC), were the snow class inputs used to produce SnowMAP. The seasonal snow classification^26^(i.e., meteorological snow classes in the text) was derived using 0.1° global meteorological climatologies of air temperature and precipitation from European Centre for Medium-Range Weather Forecasts (ECMWF) Reanalysis, 5th Generation Land (ERA5-Land), using the period from 1981 to 2019. MicroMet^55^ was used to downscale the meteorological variables to finer spatial resolutions used in the snow class products. The European Space Agency (ESA) Climate Change Initiative (CCI) GlobCover land-cover data was used to distinguish between high (non-forested) and low (forested) areas. The precipitation (Snowfall Precipitation Rate, SPR) and temperature (Cooling Degree Months, CDM) climatology datasets used in Sturm and Liston 2021^26^ to derive the seasonal snow classification were provided by Dr. Glen Liston to support this analysis. ERA5-Land monthly average data (https://cds.climate.copernicus.eu/datasets/reanalysis-era5-land-monthly-means) were also used to derive per-pixel 2-meter air temperature averages from 1981 to 2019 for all global land areas on a 0.1° x 0.1° grid^53^. To provide summaries for each SnowMAP class, this dataset was resampled to match the global 30 arc-second (1/120°) SnowMAP grid via nearest-neighbor interpolation.

The Sturm and Liston 2021^26^ classification uses multiple thresholds to partition air temperature and precipitation regimes for all global land areas. For air temperature, their algorithm uses a critical monthly mean air temperature to determine which months to include in the cumulative snow-possible season temperature metric (CDM). This threshold is set at 10 °C to allow snow covers to exist in areas where the monthly mean temperature may be above freezing (0 °C), as is common in wet maritime and ephemeral/short-lived snow cover regions. Using the calculated CDM values, areas are then partitioned into very high (< 61 °C), high (61 to 125 °C), and low (> 125 °C) temperature classes. The very high threshold of 61 °C was determined by minimizing the difference between the mapped extent of ephemeral snow and the observed extent from MODIS-derived ephemeral snow cover (< 2 months of consecutive snow cover on average, 2001–2016) in Wrzesien et al., 2019^67^. The threshold between high and cold temperatures of 125 °C was determined using experimental data from a range of observational sites across Alaska, USA. Analysis of observed snowpack and air temperature characteristics identified a breakpoint between persistent and cold snowpacks (< -10 °C for ~ 6 months) that occurred between CDM values of 100 °C and 150 °C. For precipitation, the snowfall precipitation rate (SPR) is the mean precipitation rate for only snow-possible months, again defined by the critical temperature of 10 °C. Precipitation regimes are classified as high or low using an SPR threshold of 4 mm/day. While the original classification identified a breakpoint at ~ 2 mm/day based on observational data, the threshold was adjusted upward to 4 mm/day to account for known biases in the observations and coarser-resolution precipitation products used in 1995.

The snow cover climatology was derived from daily gap-filled MODIS/Terra snow cover observations spanning 2000 to 2023. Using these records, snow persistence (i.e., snow cover duration), the core snow season (i.e., the longest sustained period of snow cover), and snow season persistence (i.e., the proportion of time with snow cover between the first and last snow cover observation) were calculated at all 0.01° global pixels for each winter. As detailed in Johnston et al. (2023)^33^, clustering combined with a decision tree framework was used to distinguish between ephemeral, transitional, and seasonal snow classes. No snow areas were defined as having no observed snow cover in a given winter, and perennial areas were defined as areas with more than 11 months of snow cover. Snow cover metrics provided in the NSIDC snow cover climatology dataset, such as the core snow season, were averaged for the 2000 to 2023 period and used to characterize snow cover conditions in this study.

#### Snow depth observations

The Global Historical Climatology Network daily dataset (GHCNd, https://www.ncei.noaa.gov/products/land-based-station/global-historical-climatology-network-daily) includes more than 50,000 sites with snow depth observations spanning from 1857 to the present day, serving as the most comprehensive ground-based global snow depth record^68^. Due to the varying continuity of these records (e.g., different start dates, data gaps) and to represent the period covered by the snow class datasets, we used only GHCNd data from stations with at least 30 years of observations in or after 1981. This resulted in the inclusion of 9,360 snow depth observational sites comprised of more than 94 million daily snow depth observations collected from sites spanning all SnowMAP classes in this study (see Supplementary Fig. 2 and Supplementary Table 3). Each included site was assigned to the nearest snow class, then all daily data within each snow class were summarized relative to the date with the lowest hemispheric snow cover extent^33^, which was defined to start on August 1st in the Northern Hemisphere and March 1st in the Southern Hemisphere. This was done to normalize the snow season across hemispheres. Fill values were omitted from the aggregated dataset, then snow depth values were summed from 0 to 500 cm using 10 cm bins. Zero values were summed in an additional bin.

#### Other geospatial variables

Several additional geospatial datasets were applied in this study to support the analysis of SnowMAP classes, referred to as snow classes below. These datasets are detailed as follows:

*Elevation*: The Global Multi-resolution Terrain Elevation 2010 (GMTED 2010) 30 arc-second (~ 1 km) dataset^66^ was used as input to the snow class combination workflow and to assess relationships between snow classes, elevation, and latitude using linear regression fits for each snow class (x = elevation, y = latitude). Elevation data is included from 90 °S to 84 °N. The data is available at: https://www.usgs.gov/coastal-changes-and-impacts/gmted2010.

*Land cover and land use*: The 2023 MODIS/Terra+Aqua Global Land Cover Yearly Version 6.1 500 m product (MCD12Q1.061) was aggregated to a 30 arc-second (~ 1 km) grid by taking the most common IGBP (17-class) cover classification of all 500 m sub-pixels within each grid cell. To ensure the dataset provided estimates for all land areas included in the snow classifications, in land areas with missing data, the nearest non-water land cover value was used. IGBP cover classes were grouped (as in Supplementary Table 5) and the water class removed, yielding 11 classes for analysis. The data is available at: https://www.earthdata.nasa.gov/data/catalog/lpcloud-mcd12q1-061.

*Forest density*: Maps of the total number of trees in each 30 arc-second grid cell were produced by Crowther et al. (2015)^69^ for global land areas between 55 °S and 84 °N. For this study, the total number of trees was converted to forest density in trees per km^2^ by dividing by the area of each grid cell. The data is available at: https://elischolar.library.yale.edu/yale_fes_data/1/.

*Population*: The 2020 WorldPop dataset^46^ provides the spatial distribution of human populations, expressed as people per grid cell, as a 30 arc-second gridded dataset covering all global land areas. For population density (people per km^2^) summary statistics, the population was summed across all grid cells within each snow class, then divided by the class area. The data is available at: https://www.10.5258/SOTON/WP00647.

*Transportation infrastructure*: The Global Roads Inventory Project (GRIP4) 5 arc-minute (~ 8 km) global road density dataset^70^ was used to characterize the density (meters per km^2^) of transportation networks, using the all road types subset. The density data were resampled to a 30 arc-second (~ 1 km) grid using nearest neighbor interpolation and were then multiplied by the area of each encompassing grid cell to get estimates of the total roadway length for each grid cell. The average roadway density was calculated by summing the total roadway distance divided by the area of each snow class. The data is available at: https://www.globio.info/download-grip-dataset.

*Ski areas*: The inventory of global ski areas and lifts^48^ was downloaded from OpenSkiMap.org as of April 2025. The dataset was filtered to include only operational ski areas, and each area was assigned to the nearest snow class based on the provided latitude and longitude coordinates. The number of areas and the downhill distance were summed by snow class. The data is available at: https://openskimap.org/?about.

*Hydrologic reference basin analysis*: We used basin boundaries from the Geospatial Attributes of Gages for Evaluating Streamflow (GAGES-II^49^) hydrologic streamflow reference dataset to identify proximal basins of similar size but with different SnowMAP snow phenology classes. We then extracted the basin IDs and metadata from the GAGES-II dataset and long-term flow records using the United States Geological Survey (USGS) ‘Surface-Water Historical Instantaneous Data for the Nation: Build Time Series’ tool. Data were downloaded for each basin for all water years (October 1 – September 30 of the following year) since the start of the MODIS record (2000). For each pair of proximal basins, only complete water years at both sites were used in the analysis. For the basins in the Pacific Northwest of the United States, this period was from October 1, 2006, to September 30, 2023 (17 water years). For the Northeastern United States basins, this period was from October 1, 2007, to September 30, 2023 (16 water years). All flow data were first aggregated to daily averages for each day in the period of record and then summarized by Julian date using the median flow across all water years. Specific discharge was calculated by dividing these median flows (in cubic meters per second) by the basin area (square kilometers). SnowMAP snow phenology classes were summarized by percent areal coverage within each basin boundary using the ‘Clip Raster by Mask Layer’ in QGIS.

## Supplementary Information

Below is the link to the electronic supplementary material.

Supplementary Material 1

## Data Availability

Both snow classification datasets used in this project are available on NSIDC. (1) The Sturm and Liston 2021 ‘Global Seasonal-Snow Classification, Version 1’ (https://nsidc.org/data/nsidc-0768/versions/1), and (2) The Johnston et al., 2023 ‘MODIS/Terra Global Annual 0.01Deg CMG Snow Cover Climatology, Version 1‘ (https://nsidc.org/data/nsidc-0791/versions/1). MATLAB scripts used to reproduce the merged SnowMAP snow classification are available on GitHub https://github.com/jjohns60/SnowMAP. The 30 arc-second (~1 km) SnowMAP global raster is available at https://doi.org/10.5281/zenodo.17373632.
