# Supplementary material for: The snow meteorology and phenology classification (SnowMAP): global snow cover observations enhance snow’s representation
Source: Sci Rep. 2026 Mar 18;16:14075. doi: 10.1038/s41598-026-44321-x (PMC13136412; doi:10.1038/s41598-026-44321-x)
Supplement: Supplementary file 1 — Supplementary Material 1 [file 41598_2026_44321_MOESM1_ESM.docx]

**Supplemental Materials for Manuscript:**

**The Snow Meteorology and Phenology Classification (SnowMAP): Global Snow Cover Observations Enhance Snow’s Representation**

Jeremy Johnston^1^, Jennifer M. Jacobs^1,2^, Megan Vardaman^2^, Eunsang Cho^3^

^1^Earth Systems Research Center, University of New Hampshire, Durham, NH, USA

^2^Department of Civil and Environmental Engineering, University of New Hampshire, Durham, NH, USA

^3^Ingram School of Engineering, Texas State University, San Marcos, TX, USA

**Supplementary Table 1. Thresholds that are used to define the specified snow classifications.**

**
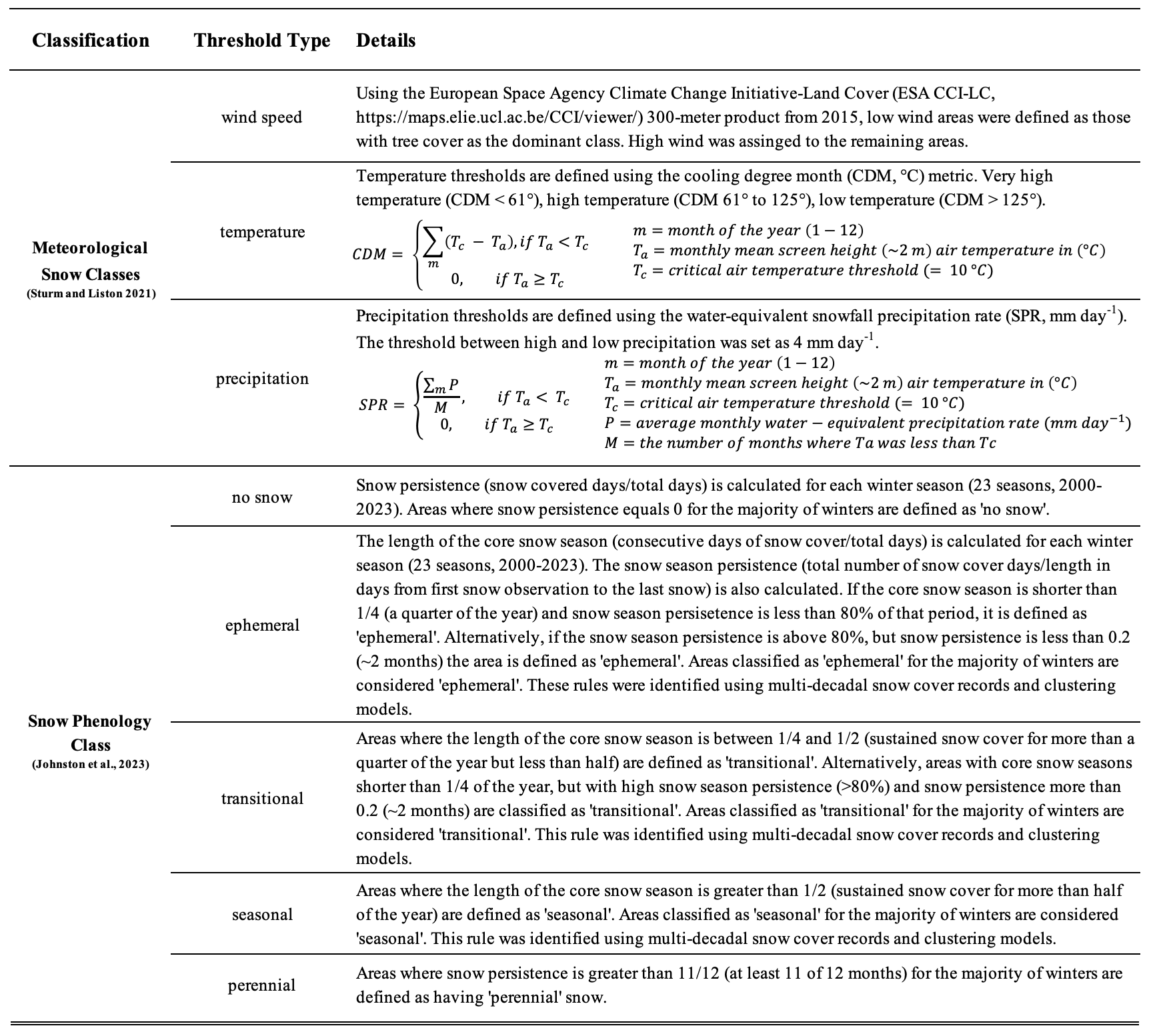
**

**Supplementary Figure 1. Asia, North America, and Europe encompass a range of snow classes, whereas Africa, South America, Australia, and Oceania are generally snow-free.** SnowMAP class spatial extent by continent in square kilometers (km^2^). Ordered left to right, from the largest to the smallest continent.

**Supplementary Table 2.** Meteorological (1981–2019 via Sturm and Liston 2021 and ERA5-Land) and snow cover (2000–2023 via Johnston et al., 2023) summary metrics for the specified SnowMAP class. The average 2-meter air temperature is calculated per-pixel (0.1°) using all average monthly temperature products from ERA5-Land between 1981 and 2019. The percentage (%) of tall vegetation is derived from the 2018 European Space Agency Climate Change Initiative (ESA CCI) 300-meter resolution land cover products, using the methodology from Sturm and Liston (2021) to partition between tall (forests) and short (or no) vegetation. SNOWMAP classes are derived on a 30 arc-second (~1 km) global grid. For a given location and winter (i.e., snow season), snow cover duration refers to the total number of snow-covered days, core snow season length refers to the maximum number of continuously snow-covered days, and snow season persistence refers to the proportion of the winter that is snow-covered between the first and last date of observed snow cover.


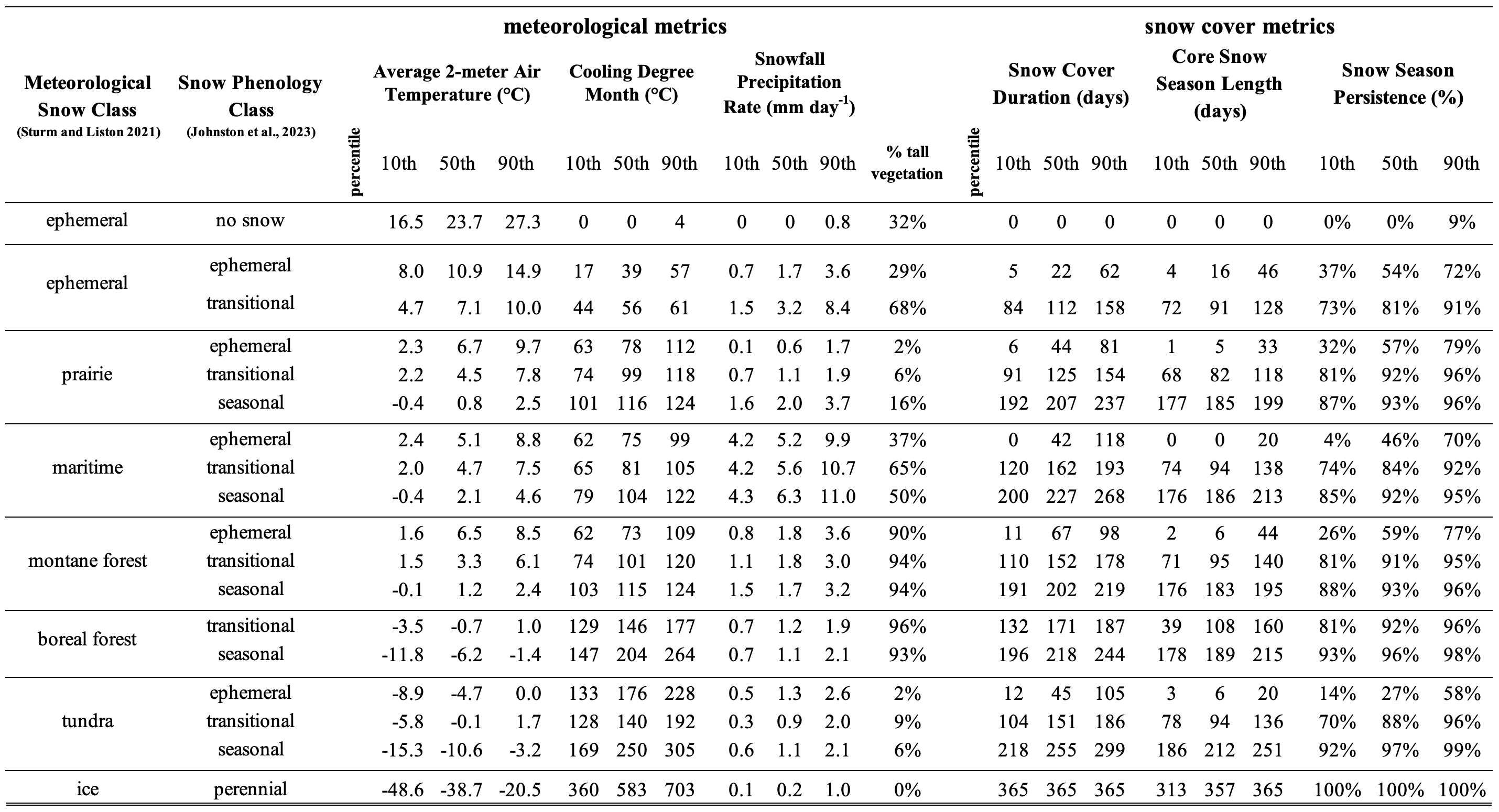


**Supplementary Figure 2. Global Historical Climatology Network (GHCN) stations included in this study.** The global distribution of 9,360 GHCN stations that include at least 30 years of snow depth data in or after 1981. Areas with solid coloring indicate areas of dense station coverage. Some stations may be obscured.

**
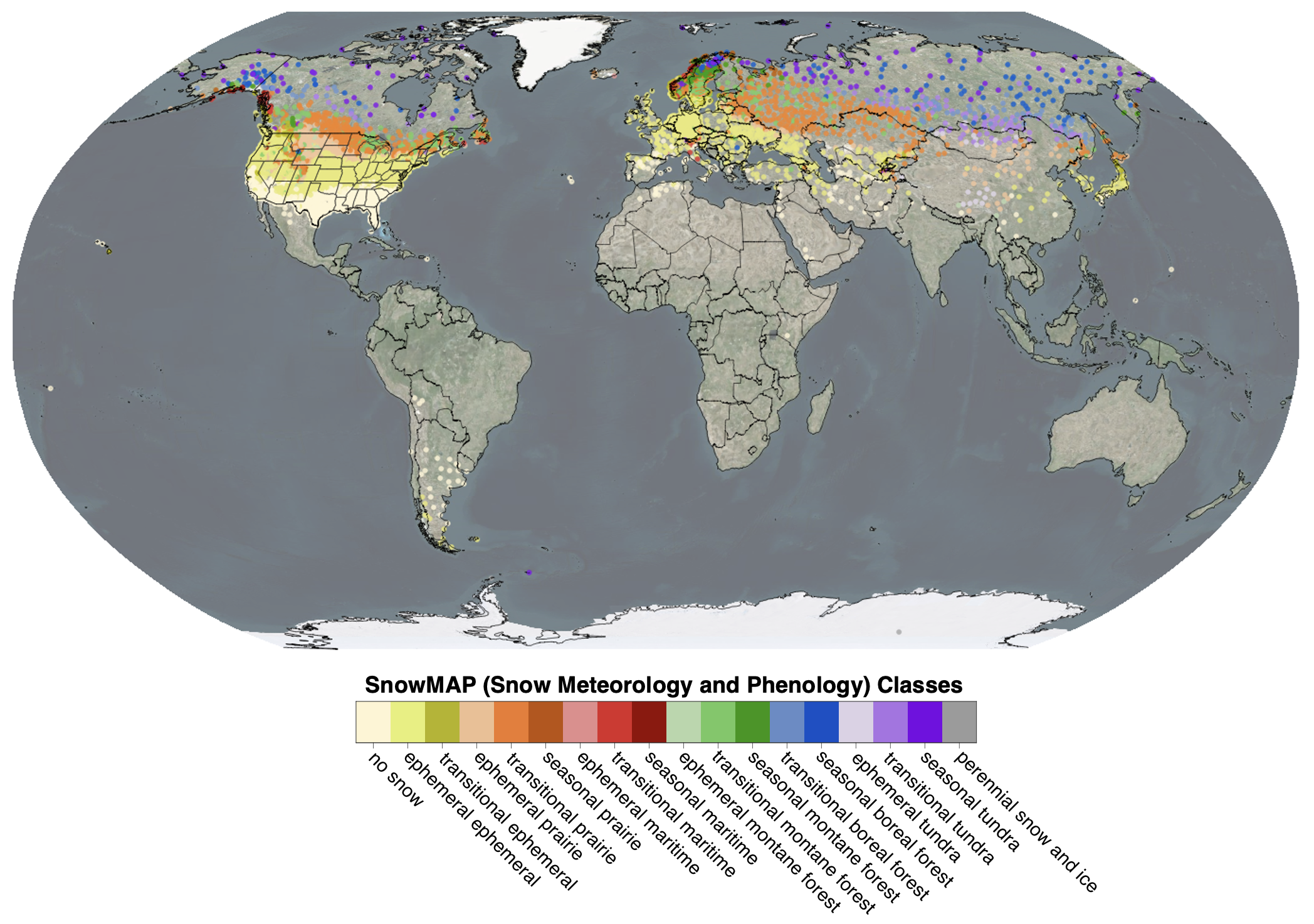
**

**Supplementary Table 3.** Summary of Global Historical Climatology Network (GHCN) daily snow depth observations from 1981 to 2025 by SnowMAP class. Peak snow depths and the corresponding dates, calculated using a 2-week moving average, are presented for the Northern Hemisphere (NH). The lower bound (2.5^th^ percentile), median (50^th^ percentile), and upper bound (97.5^th^ percentile) of all observations are shown for each seasonal period. Lower-bound peak depths are excluded because there is no distinct lower-bound peak for most snow classes. Months corresponding to the seasonal periods by hemisphere are included (Southern Hemisphere, SH). The color gradient ranges from no snow (0 cm, white) to very deep snow (> 500 cm, dark blue).


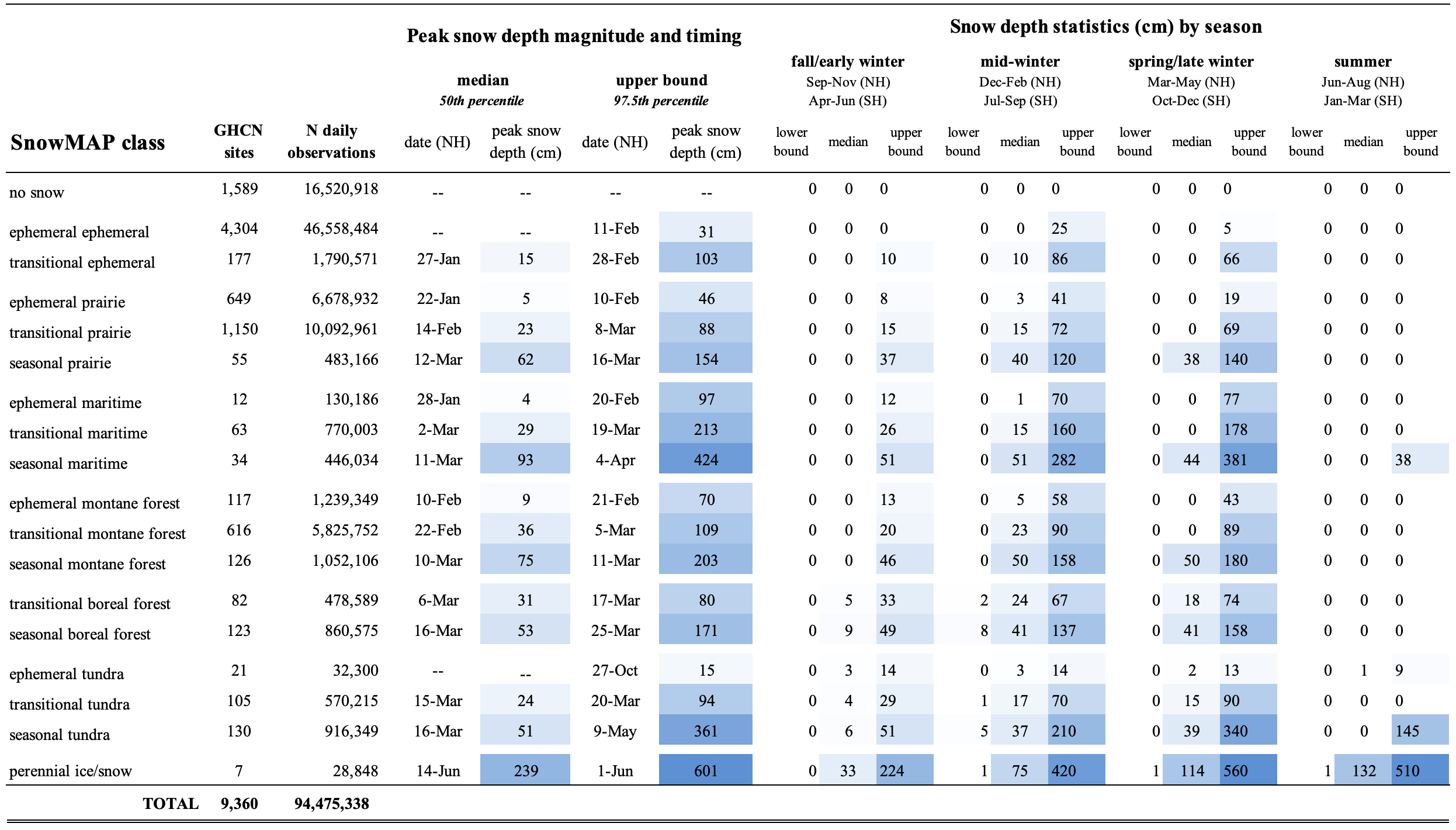


**Supplementary Table 4.** Linear regression fit statistics for the relationship between elevation in kilometers (x, km) and the absolute latitude (y, °) at which SNOWMAP classes are observed. The no snow and perennial snow/ice SNOWMAP classes are excluded. The slope defines the decrease in degrees latitude at which a given SNOWMAP class could develop for each 1 km (1000 m) increase in elevation.


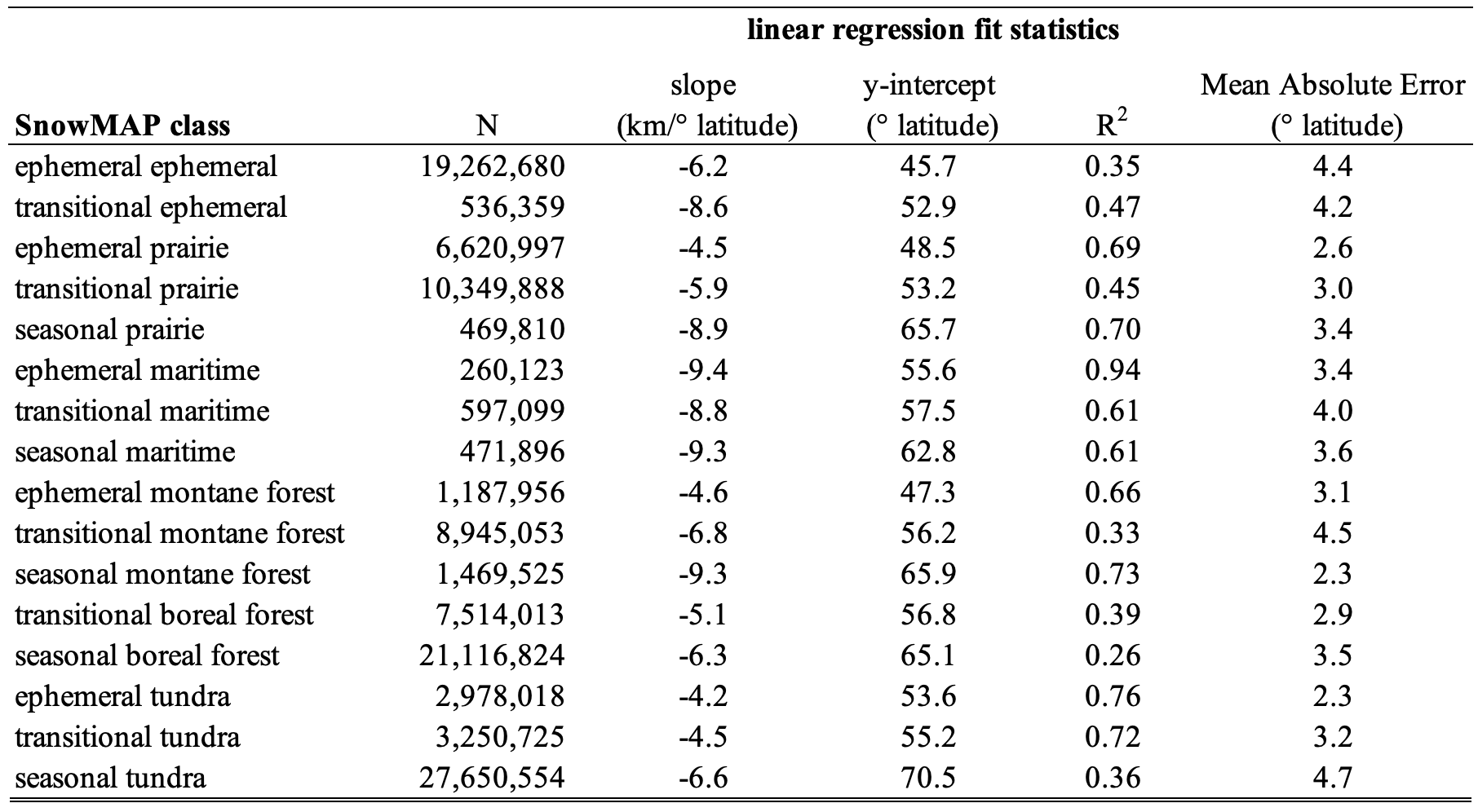


**Supplementary Table 5.** Proportion of specified land cover class (using aggregated MODIS IGBP land cover classes) within each SNOWMAP class. The most commonly occurring MODIS (~500 m) land cover class (‘majority’) is taken for each SNOWMAP pixel (~1 km) to match products to the same grid. Each row sums to 1 (100%).

**
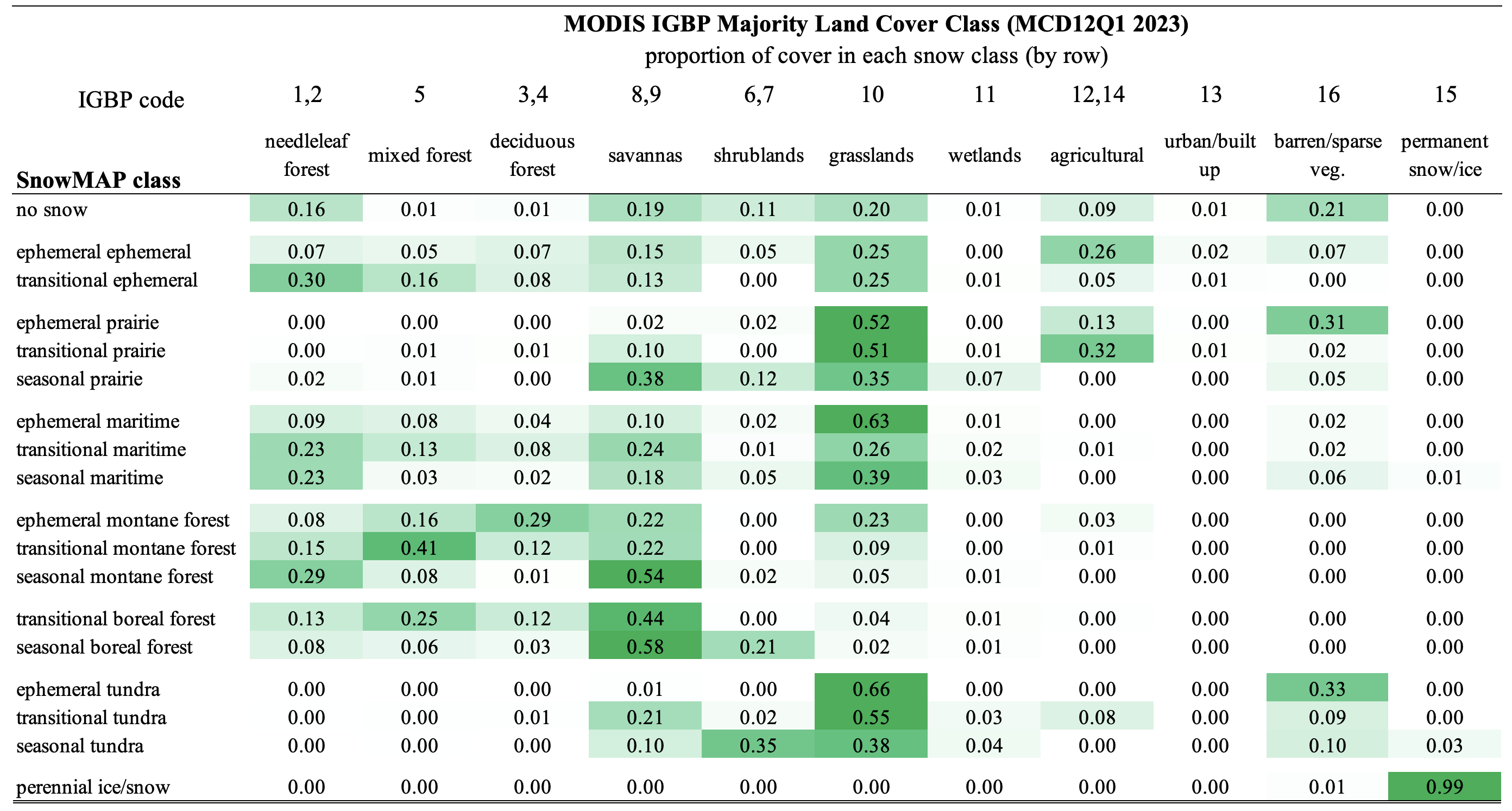
**

**Supplementary Table 6.** Global populations by SnowMAP class. Statistics are calculated for each class using the Worldpop 2020 dataset. Populations in unclassified areas in SNOWMAP, generally adjacent to large water bodies or on small islands, account for 1.0% of the global population and are not included in the table.

**
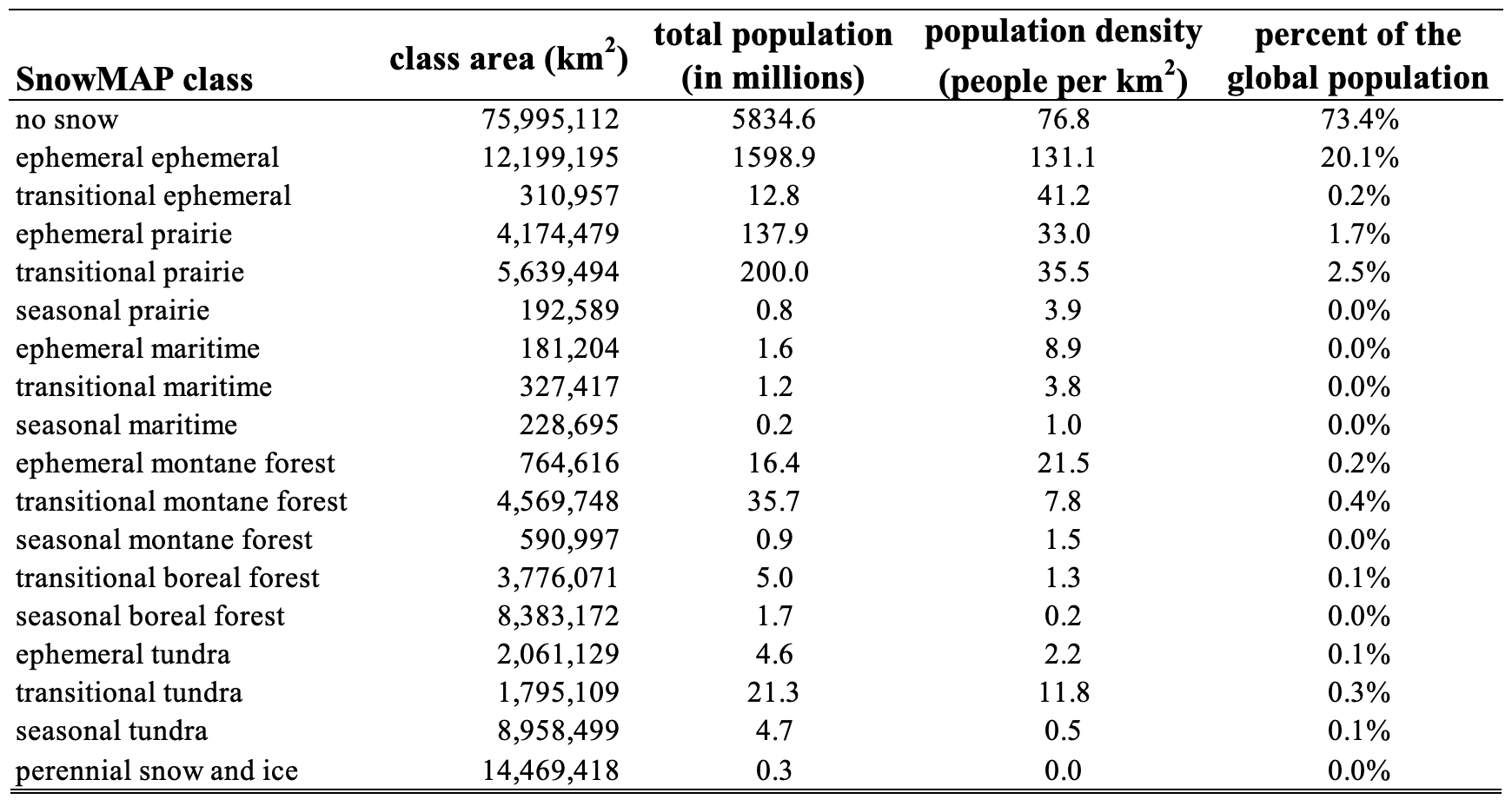
**
